# Socio‐demographic and geographic disparities in HIV prevalence, HIV testing and treatment coverage: An analysis of 108 national household surveys in 33 African countries

**DOI:** 10.1002/jia2.70024

**Published:** 2025-08-13

**Authors:** Adrien Allorant, Salome Kuchukhidze, James Stannah, Yiqing Xia, Sanele S. Masuku, Gatien K. Ekanmian, Jeffrey W. Imai‐Eaton, Mathieu Maheu‐Giroux

**Affiliations:** ^1^ Department of Social Statistics and Demography University of Southampton Southampton UK; ^2^ Center for Communicable Disease Dynamics Department of Epidemiology Harvard T.H. Chan School of Public Health Boston Massachusetts USA; ^3^ United Nations Program on HIV/AIDS (UNAIDS) Johannesburg South Africa; ^4^ Department of Epidemiology Biostatistics, and Occupational Health McGill University Montréal Quebec Canada; ^5^ MRC Centre for Global Infectious Disease Analysis School of Public Health, Imperial College London UK

**Keywords:** 95‐95‐95, Bayesian statistics, equity, HIV/AIDS, HIV care cascade, sub‐Saharan Africa

## Abstract

**Introduction:**

Socio‐demographic and geographic disparities in HIV prevalence, uptake of HIV testing and access to antiretroviral therapy (ART) persist in high HIV burden countries. Understanding demographic, spatial and temporal factors can guide interventions.

**Methods:**

We analysed 108 geo‐referenced population‐based surveys conducted over 2000–2023 across 33 African countries, involving 2.3 million respondents. Multilevel Bayesian logistic regression models assessed associations between HIV outcomes (HIV prevalence, recent HIV testing and ART coverage) and socio‐demographic characteristics (age, education, place of residence, relative wealth), geographic location (country, district) and time trends. Separate models were estimated for men and women in central, eastern, southern and western Africa.

**Results:**

Inequalities in HIV risk and access to testing and treatment services were driven by differences in educational attainment and within‐country variations. In southern Africa, women with tertiary education had a 12%‐point lower HIV prevalence (95% Credible Interval [CrI]: −27% to −2%) than those with less than primary education. In eastern Africa, they had a 13%‐points (95% CrI: 2−22%) higher probability of recent HIV testing. Associations with relative wealth were weaker and more heterogeneous: in southern Africa, HIV prevalence shifted over time from higher to lower wealth quintiles, and adolescent girls and young women became the most frequently tested age group. In central Africa, wealthier men maintained higher recent testing and ART coverage levels. District‐level variations accounted for disparities in HIV outcomes. In western Africa, the expected difference in ART coverage between individuals with similar socio‐demographic characteristics living in different districts was 14%‐points (95% CrI: 3−32%) for men and 10%‐points (95% CrI: 3−27%) for women.

**Conclusions:**

Disparities in HIV outcomes are strongly associated with differences in education, and across districts of the same country. Higher education levels are associated with lower HIV prevalence, greater testing and higher ART coverage, while districts with limited services sustain higher population viraemia. Despite the scale‐up of HIV prevention and treatment programmes, important disparities remain, and renewed education‐centred and geographically targeted efforts are needed to close gaps.

## 1 INTRODUCTION

Eastern, southern, western and central Africa have the highest HIV burden globally, with an estimated 640,000 new acquisitions and 390,000 AIDS‐related deaths in 2023 [1]. HIV acquisition and transmission risks, however, vary substantially along geographic, age, gender, education and wealth dimensions [2, 3]. Addressing health inequalities [4] is reflected in the health‐related Sustainable Development Goals (SDG 3 and 5) and the *Global AIDS Strategy 2021–2026* [5, 6]. Mitigating inequalities in access to HIV prevention and treatment services is critical because disparities can exacerbate the risk of HIV acquisition and transmission; when interventions fail to reach specific groups, inequalities are perpetuated and can become further concentrated over time [7, 8].

Substantial within‐country heterogeneity in HIV prevalence was recognized early in the HIV epidemic [9, 10]. Later, in the 2010s, focusing HIV programmes on highest burden locations to maximize impacts catalysed numerous spatial analyses aimed at assessing inequalities in HIV prevalence and access to HIV testing and treatment [11]. This motivated improved data and methods for subnational analyses [12, 13, 14]. Important data sources include Demographic and Health Surveys (DHS), which typically include approximate Global Positioning System (GPS) coordinates of surveyed communities and collect data on HIV prevalence and HIV service uptake, and, since 2015, Population‐based HIV Impact Assessment (PHIA) surveys, which also contain behavioural and bio‐markers, enabling comprehensive assessment of HIV epidemics.

Research has extensively investigated the role of socio‐demographic factors, such as age, gender, location of residence, education and wealth, in influencing HIV/AIDS epidemics in Africa [15, 16, 17, 18]. Gender and age disparities are particularly stark, with adolescent girls and young women disproportionately at risk of HIV acquisition [19], while men living with HIV—particularly younger men—having lower engagement in testing and treatment [20, 21, 22, 23, 24]. In contrast, evidence on the impact of socio‐economic status (SES) on HIV‐related outcomes is more varied. Early analyses of HIV seroprevalence surveys found that higher educational attainment [25] or wealth [26] was associated with higher HIV prevalence. Recent studies provided mixed results: one multi‐country analysis found evidence of both inequalities favouring individuals with higher or lower SES, depending on the country, while a systematic review reported that the effect of wealth was imprecise, but that higher educational attainment was associated with engagement in HIV care [27, 28]. Considering heterogeneity in study methods, it remains unclear whether contrasting results are due to methodological differences or indicative of shifts over time.

Despite attention to subnational disparities and extensive literature on individual‐level risk factors, few analyses have simultaneously considered geographic and individual‐level factors when examining inequalities. Yet, spatial factors can distort the relationships between socio‐demographic variables and HIV prevalence, testing, and treatment. Utilizing joint analyses of the effects of time, space and socio‐demographic determinants, our study addresses this challenge by fitting Bayesian small area models to all available georeferenced population‐based survey data on HIV prevalence, and HIV testing and treatment services, since 2000 in 33 African countries.

## 2 METHODS

### 2.1 Data

We analysed nationally representative population‐based surveys conducted in African countries between 2000 and 2023 that included geographic information on survey clusters and collected data on at least one of the following indicators: (1) HIV serostatus; (2) self‐reported HIV testing (and receipt of results) in the 12 months before the survey interview; or (3) antiretroviral therapy (ART) coverage (either self‐reported ART use or detection in blood samples), among people living with HIV (PLHIV). These three indicators are critical for appraising inequalities in HIV risk and uptake of services [3, 29]. HIV prevalence is a measure of the epidemic's burden, HIV testing is an entry point to both care (for PLHIV) and prevention strategies for those who test negative, and ART coverage is a measure of the reach of HIV services among PLHIV. The main survey series meeting these criteria were the DHS and the AIDS Indicator Survey (AIS), PHIA, South African HIV Behavioural, Sero‐status and Media (SABSSM), the Nigeria AIDS Indicator and Impact Survey (NAIIS) and the Botswana HIV/AIDS Impact Survey (BAIS)—which are described elsewhere [30, 31, 32, 33].

### 2.2 Statistical analyses

Each outcome of interest—HIV prevalence, HIV testing in the past 12 months (henceforth “recent HIV testing”) and ART coverage among PLHIV—was modelled separately using a space‐time logistic regression model that incorporated the corresponding sampling weights. For data preparation, weighted counts were calculated for each outcome using the Kish effective sample size, stratified by survey region and urban/rural location, ensuring both numerators and denominators accounted for unequal sampling probabilities [34]. Weighted counts were tabulated by second administrative subdivisions (henceforth referred to as “districts”), year, age group (15−24, 25–34, 35–49, ≥50 years old), sex, educational attainment (less than primary education; primary education; secondary education; tertiary education), place of residence (urban; rural) and households’ relative wealth quintile, which was calculated by obtaining a weighted frequency distribution of households’ wealth score stratified by place of residence (see Supplementary Material 1, and Figure S1.1 for details). We compared a sequence of five multilevel Bayesian logistic regression models for each outcome, by region (e.g. eastern, central, southern or western Africa [35]), country, district and socio‐demographic groups, across time. The five models were of increasing complexity; all included random intercepts for district and country, fixed effects for age, place of residence, education and relative wealth, and a first‐order autoregressive process for calendar year (Model 1). To allow for different temporal trends by district and country, we introduced random slopes via district‐year and country‐year interaction terms (Model 2). To account for the possibility that the influence of socio‐demographic covariates may have changed over the study period, we included product terms between time period (2003−2007, 2008–2012, 2013–2017, 2018–2023) and age, place of residence, educational attainment and relative wealth (Model 3). Finally, Models 4 and 5 were identical to Models 2 and 3, respectively, but used spatially correlated rather than independent district random effects, permitting correlation between neighbouring districts. The best‐performing model for each outcome was identified by calculating the models’ goodness of fit and complexity indicators (Log‐Conditional Predictive Ordinate [LCPO], Deviation Information Criteria [DIC], Widely Applicable Information Criteria [WAIC]). Due to computational constraints and to allow heterogeneity in the effect of socio‐demographic covariates, spatial structures and temporal trends, we fitted separate models by sex and region. All models used weakly informative normal priors for the fixed effects and penalized‐complexity priors on the precisions of the random effects (see Supplementary Material 1).

We reported measures of association by calculating the average predictive comparisons (APCs) for each category of each covariate across different years and countries. APCs measure the expected difference in the probability of the outcome (e.g. having received an HIV test in the past 12 months) when changing one variable's category (e.g. achieved secondary education) to another category of the same covariate (e.g. less than primary education), with all other covariates constant at their observed values [36]. APCs are preferred to odds ratios because they provide effect estimates directly interpretable on the outcome scale in terms of “predicted change in probability,” and because our models include interactions, which preclude the interpretation of individual odds ratios as comparisons “holding all other variables constant” [37]. Following Howes et al. [38], we calculated the proportion of the total variation explained by each model component at various levels of the model's hierarchy.

We conducted sensitivity analyses to examine the impact of including both education and relative wealth in the models, as the impact of education could be partially mediated by relative wealth (or vice versa). Specifically, we fitted models excluding education to observe how the coefficients for relative wealth changed, and models excluding wealth to observe how the coefficients for education changed.

### 2.3 Ethics

This study involved secondary analysis of publicly available upon request, de‐identified data. The DHS, PHIA, AIDS, SABSSM and BAIS received informed consent for participation and HIV testing from all participants at the time of data collection, and obtained ethical approval from the relevant national ethics committees’ Institutional Review Boards (IRB). No additional informed consent was required. The study protocol was reviewed and approved by the McGill University Faculty of Medicine IRB (A10‐E72‐17B).

## 3 RESULTS

**Figure 1:**
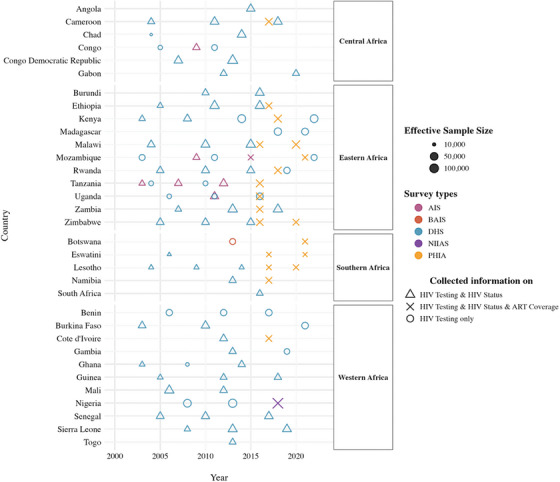
**Data availability by country and year, 2000–2023**. Points represent geolocated population‐based surveys conducted in sub‐Saharan Africa from 2000 to 2023. Circles represent surveys collecting information on recent HIV testing only; triangles represent surveys that included information on both recent HIV testing and HIV status, while crosses represent surveys that collected information on all three indicators—recent HIV testing, HIV status and anti‐retroviral treatment among people living with HIV. AIS, AIDS Indicator Survey; DHS, Demographic and Health Surveys; PHIA, Population‐based HIV Impact Assessment. Country‐specific surveys include South African HIV Behavioural, Sero‐status (SABSSM) and Botswana Aids Survey (BAIS).

We identified 108 georeferenced population‐based HIV testing surveys conducted across 33 countries between 2000 and 2023, with 2.3 million respondents. Of these, 25 surveys from 14 countries collected information on HIV testing only (562,230 respondents), while 61 surveys across 29 countries collected HIV testing history and HIV seroprevalence (1.1 million respondents). The remaining 22 surveys from 17 countries gathered information on HIV seroprevalence, HIV testing among all participants (638,450 respondents) and ART coverage (54,900 PLHIV) (Figure 1). We present APCs averaged over all years derived from the best‐performing model for each outcome (for estimated odds ratios along with APCs by period, see Figures S2.2−2.7). Table 1 summarizes the best‐performing models for each outcome across regions and sexes, based on goodness‐of‐fit and model complexity indicators (for models’ performances, see Figures S2.8−2.13). Generally, Model 3 demonstrated superior performance in modelling HIV prevalence and recent HIV testing. However, for ART coverage, the optimal model varied across different regions and sexes.

**Table 1:** Identified best‐performing models according to goodness of fit and model complexity indicators, for each outcome, by region and sex

Region	Outcome	Best model—men	Best model—women
Central Africa	HIV prevalence	Model 3	Model 3
	Recent HIV testing	Model 3	Model 3
	ART coverage	Model 3	Model 5
Eastern Africa	HIV prevalence	Model 3	Model 3
	Recent HIV testing	Model 3	Model 5
	ART coverage	Model 3	Model 2
Southern Africa	HIV prevalence	Model 3	Model 3
	Recent HIV testing	Model 3	Model 3
	ART coverage	Model 3	Model 4
Western Africa	HIV prevalence	Model 1	Model 3
	Recent HIV testing	Model 3	Model 3
	ART coverage	Model 2	Model 4

*Note*: Supplementary Material 1 Methods section includes a full description of the five model formulations tested, and in Supplementary Material 2, we display the values of the criteria used (Widely Applicable Information Criterion [WAIC], Deviance Information Criterion [DIC], log Conditional Predictive Ordinate [LCPO]) to select the best fitting models (Figures S2.8–S2.12). All models included random intercepts for district and country, fixed effects for age, place of residence, education and relative wealth, and a first‐order autoregressive process for calendar year. Model 2 introduces random slopes via district‐year and country‐year interaction terms. Model 3 includes interactions by period (2003–2007, 2008–2012, 2013–2017, 2018–2023) for the effect of age, place of residence, educational attainment and relative wealth. Models 4 and 5 are identical to Models 2 and 3, respectively, but use spatially correlated rather than independent district random effects. See Table S1.1 for the complete list of model equations.

### 3.1 HIV serostatus

**Figure 2:**
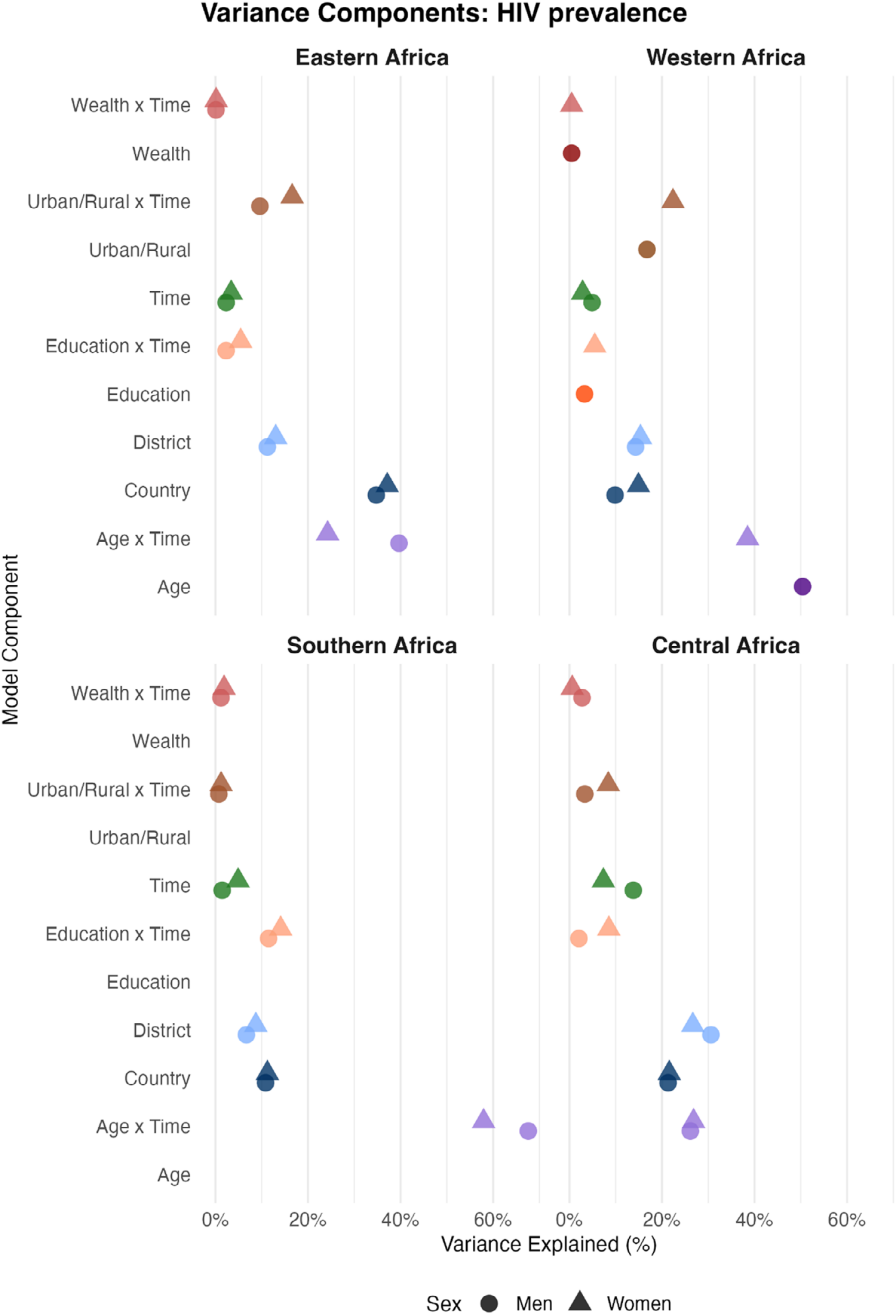
**Proportion of variance explained by each component of the best regression model for HIV prevalence**. The figure presents the proportion of total variance in HIV prevalence explained by each component in sex‐ and region‐stratified best‐performing regression models.

**Figure 3:**
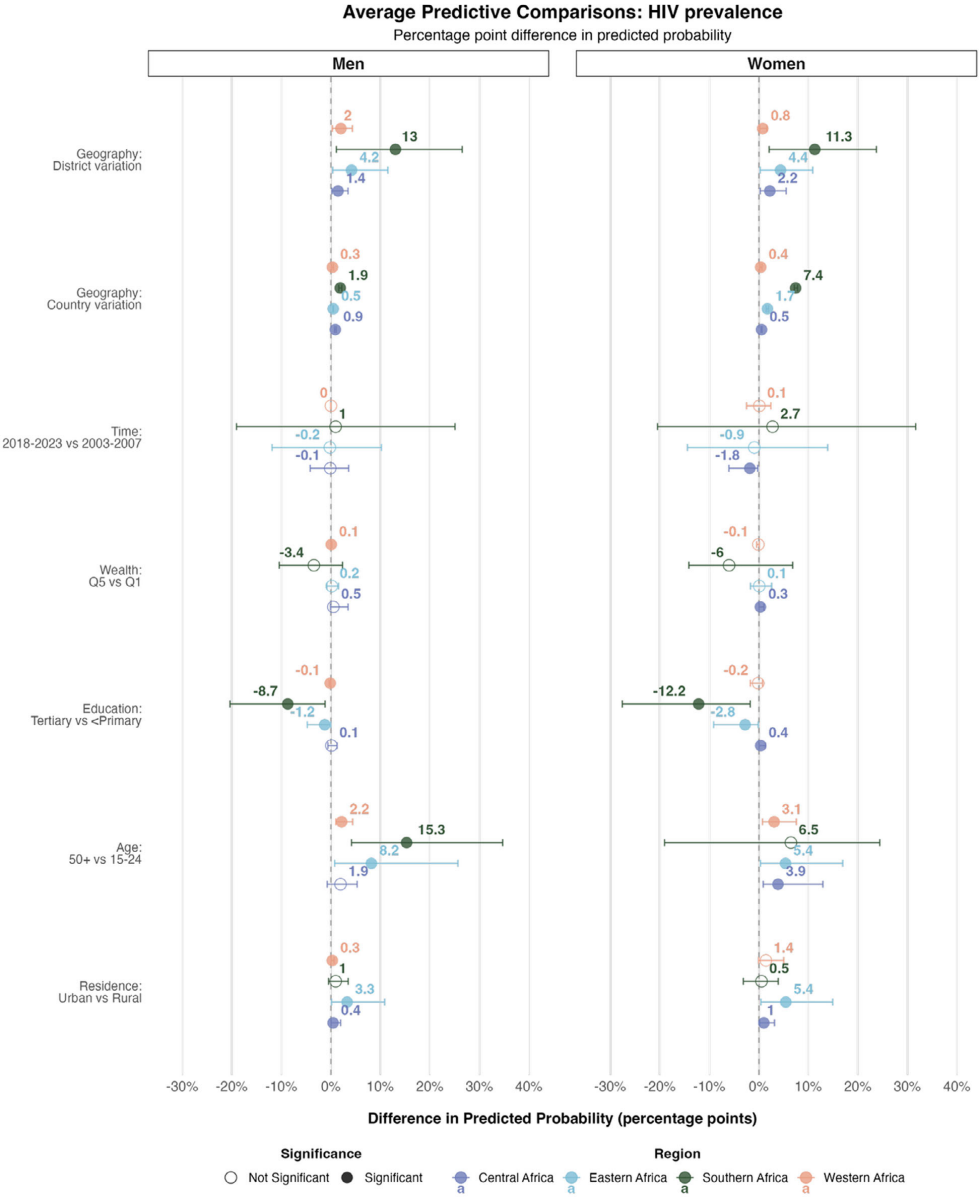
**Expected difference in the probability of living with HIV among all men and women, in sub‐Saharan Africa given a change in the covariates**. This figure shows the average predictive comparisons for the spatial terms—country and district effects—the time period effect and the socio‐demographic covariates. These are estimates of the expected difference in the probability of living with HIV between two respondents with different values for one covariate but similar values for all others, obtained by post‐estimation simulation from the model. The mean effect estimates are displayed above the circles. Horizontal lines are 95% credible intervals. Empty circles indicate estimates whose credible intervals include zero. The model was stratified by regions of Africa, which are represented on the plot with different colours.

Age consistently ranked among the two largest sources of variation in HIV serostatus in all regions, accounting for 24% (eastern African women) to 68% (southern African men) of the total variation (Figure 2). Even in the few cases where country‐ or district‐level effects were marginally higher—for example Eastern‐African women, where country accounted for 27% versus age 24%, or Central‐African men, where district accounted for 31% versus age 26%—age remained a close second (Figure S2.1 plots the top three components for every region–sex combination). The APCs comparing 35–49 and 15‐ to 24‐year‐olds was, for instance, 24.4%‐points (95% CrI: 9.7−31.9%) and 25.3%‐points (95% CrI: 4.4−46.8%), among men and women in Southern Africa, respectively (Figure 3).

The second most influential predictors of HIV serostatus differed by region. In southern Africa, education was the second most influential predictor; differences in education explained 12% of the variation among men and 14% among women. Women with tertiary education had an expected probability of HIV seropositivity that was 12.1%‐points lower (95% CrI: −27.1% to −1.7%) compared to women with less than primary education, while the expected difference for men was 8.7%‐points (95% CrI: −20.3% to −1.2%). In eastern Africa, the APC was 2.8%‐points lower (95% CrI: −9.2% to −0.1%) for women with tertiary education and 1.2%‐points lower (95% CrI: −4.8% to −0.1%) for men. In eastern Africa, country was the second most important factor, with country‐level differences explaining 35% of the variation among men and 37% among women. In western Africa, place of residence accounted for the second largest proportion of variation, with 17% among men and 22% among women. In contrast, relative wealth explained less than 1% of the variation in any region for both men and women.

Effect estimates were similar when stratifying APCs by period (Figure S2.2), except for relative wealth in southern Africa, where we observed a notable reversal in the wealth gradient over time when not averaging over the study periods: for 2008–2013, there was a clear positive gradient, with higher expected HIV prevalence among wealthier individuals, while over 2013–2017, and 2018–2023, this gradient was reversed, with higher expected HIV prevalence among poorer individuals (Figure S2.2.D).

### 3.2 Past‐year HIV testing

**Figure 4:**
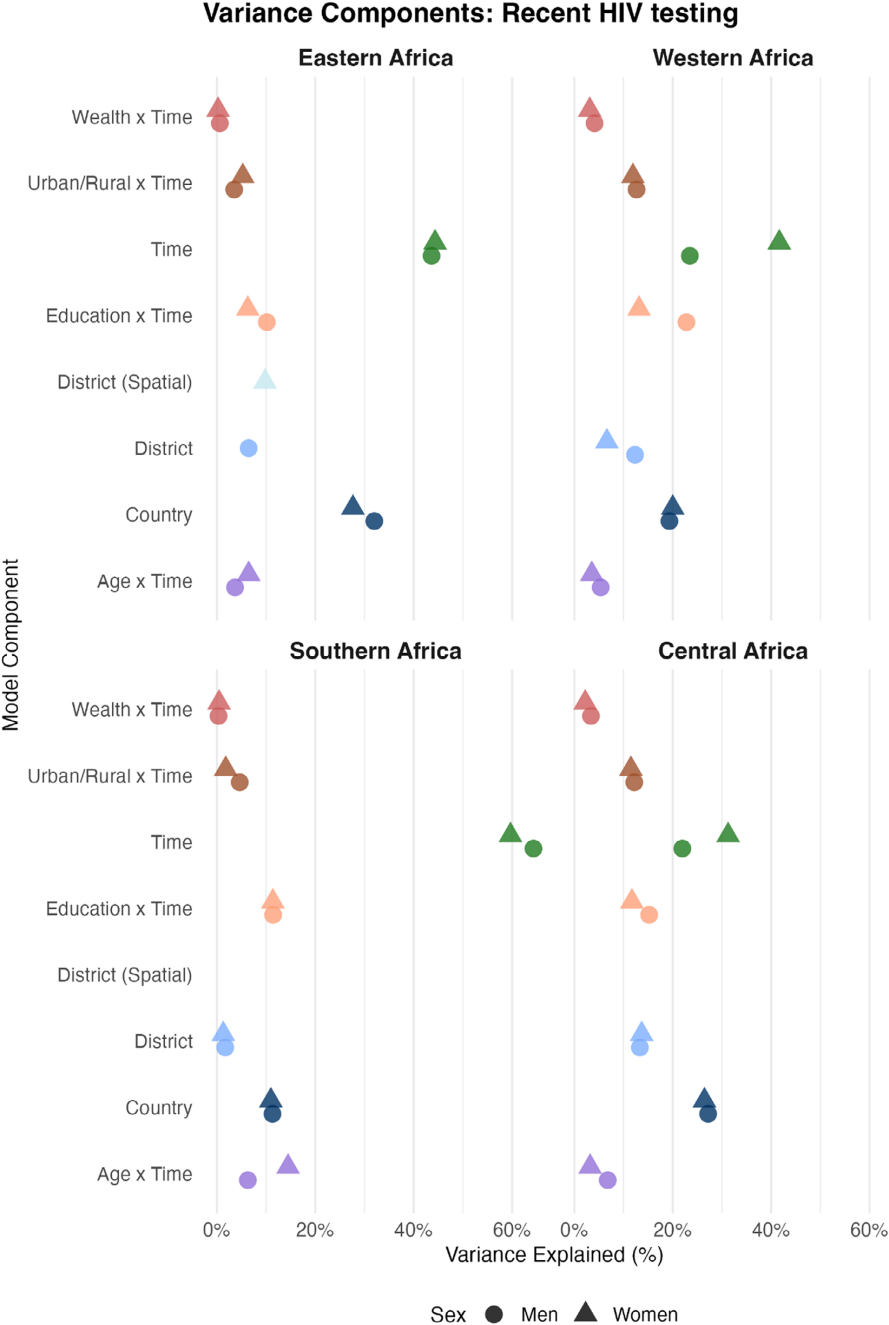
**Proportion of variance explained by each model component for recent HIV testing in sex‐ and region‐stratified models**. Layout and colour coding are identical to those used in Figure 2.

**Figure 5:**
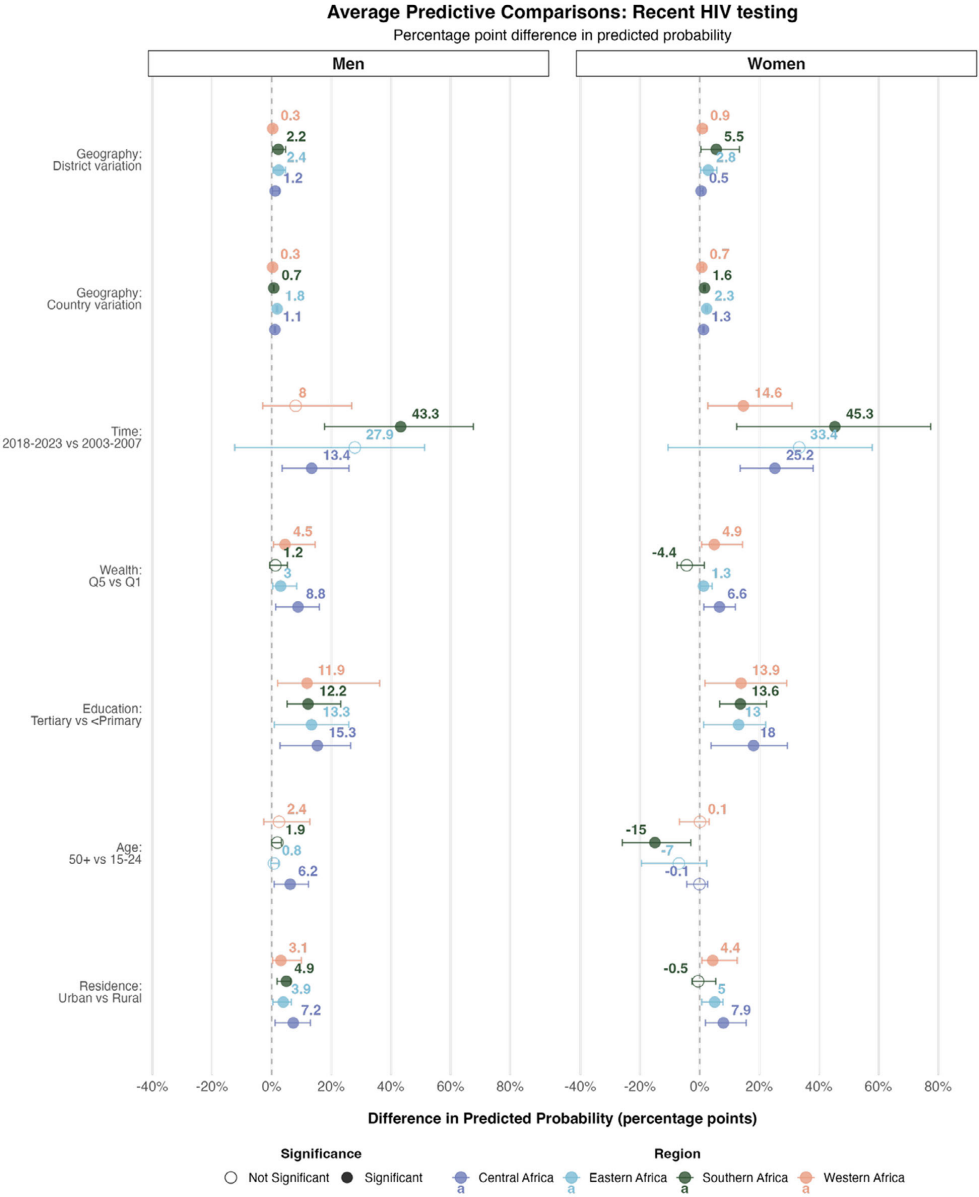
**Expected difference in the probability of recent HIV testing among all men and women, in Africa given a change in the covariates**. This figure shows the average predictive comparisons for the spatial terms—country and district effects—the time period effect and the socio‐demographic covariates. These are estimates of the expected difference in the probability of recent HIV testing (<12 months) between two respondents with different values for one covariate but similar values for all others, obtained by post‐estimation simulation from the model. The mean effect estimates are displayed above the circles. Horizontal lines are 95% credible intervals. Empty circles indicate estimates whose credible intervals include zero. The model was stratified by regions of Africa, which are represented on the plot with different colours.

Temporal changes were the leading source of variation in recent HIV testing across most regions and sex strata, accounting for 24% of the variation among men and women in western Africa, and up to 64% (men) and 60% (women) in southern Africa (Figure 4). A notable exception was among men from central Africa, where country‐level effects accounted for slightly more variation than the time effect, 27% versus 22%. The temporal period component, capturing a major proportion of the variance, is consistent with the rapid expansion of testing services over the past two decades [20, 39]. Country‐level differences were the next‐largest source of variation in central and eastern Africa. In southern and western Africa, education was the second most influential predictor, explaining 11% of the variation among both men and women in southern Africa, and 23% (men) and 13% (women) in western Africa. The expected difference in the probability of recent HIV testing between a man with tertiary education compared to a similar man with less than primary education was 12.2%‐points (95% CrI: 5.2−22.9%) and 12.0%‐points (95% CrI: 1.2−32.3%) in southern and western Africa, respectively (Figure 5). Education‐level differences also contributed substantially to variations in HIV testing uptake in eastern and central Africa, and among women, with APCs comparable to those for men. Urban residence and higher relative wealth were associated with large increases in the probability of past‐year HIV testing compared to rural residence and lower relative wealth, with estimated APCs of 7.2% (95% CrI: 1.2−13.0%) and 8.9% (95% CrI: 1.4−16.1%) among men and 7.9% (95% CrI: 2.0−15.5%) and 6.6% (95% CrI: 1.5−11.9%) among women, respectively, in Central Africa. Country and district‐level differences explained a substantial proportion of variation in past‐year HIV testing in central, western and eastern Africa, but less than 2% of the variation in southern Africa, indicating little change in recent HIV testing between districts and countries.

APCs were relatively stable over time periods (Figure S2.3), except in southern Africa, where the relationship between age‐group and recent HIV testing reversed over the study period, from adolescent girls and young women being least to most likely to have recently tested for HIV compared to all other age‐groups (Figure S2.3.A). Figure S2.3.C shows that each incremental increase in educational attainment is associated with a substantial increase in the probability of recent HIV testing.

### 3.3 ART coverage among PLHIV

**Figure 6:**
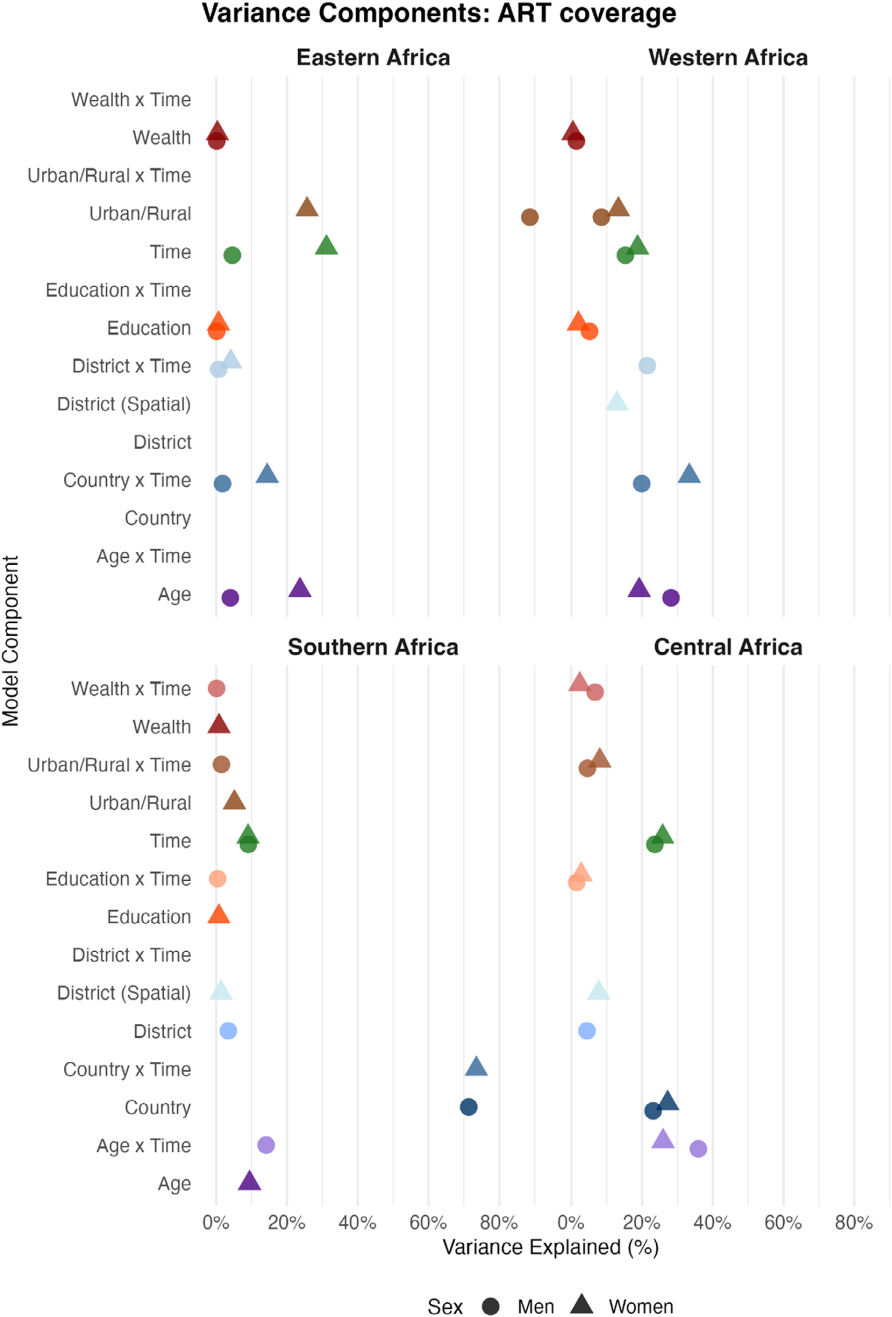
**Proportion of variance explained by each component of the regression model for ART coverage**. See Figure 2 for further details.

**Figure 7:**
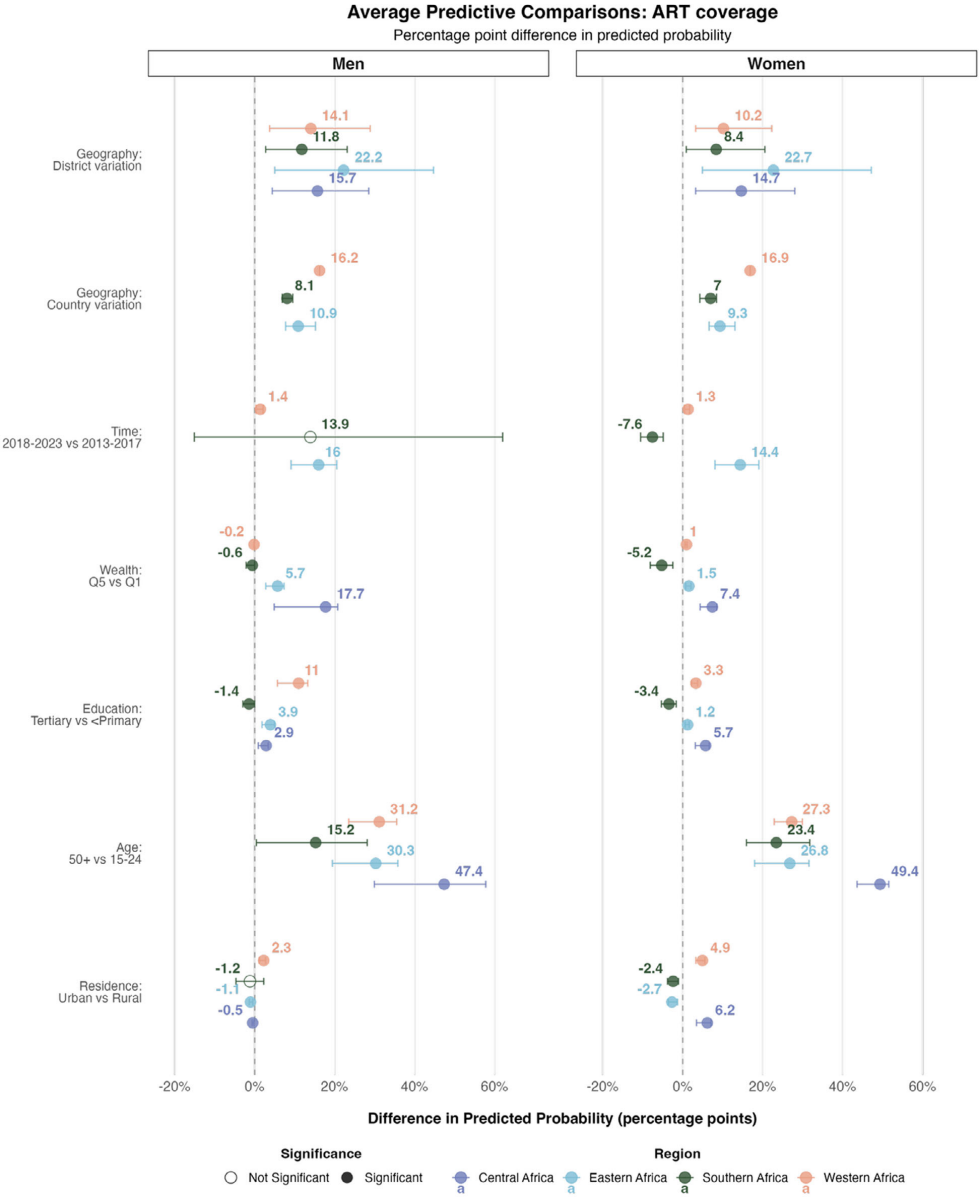
**Expected difference in the probability of ART coverage among all men and women living with HIV, in sub‐Saharan Africa given a change in the covariates**. This figure shows the average predictive comparisons for the spatial terms—country and district effects—the time period effect and the socio‐demographic covariates. These are estimates of the expected difference in the probability of ART coverage between two respondents living with HIV with different values for one covariate but similar values for all others, obtained by post‐estimation simulation from the model. The mean effect estimates are displayed above the circles. Horizontal lines are 95% credible intervals. Empty circles indicate estimates whose credible intervals include zero. The model was stratified by regions of Africa, which are represented on the plot with different colours.

The factors explaining the largest proportion of variance in ART coverage among PLHIV varied by region (Figure 6). In eastern Africa, temporal changes explained the largest proportion of variance among men (42%) and women (31%), in line with the substantial scale‐up of ART programmes in the whole region over the last two decades [1]. The APC for the period 2018–2023 versus 2013–2017 among women was substantial, with an expected increase of 14.4%‐points (95% CrI: 8.3−19.4%, Figure 7). Place of residence accounted for 26% of the variance among women, with urban women having a 2.6%‐points (95% CrI: −3.8% to −1.1%) lower probability of being on ART compared to rural women. A similar APC for place of residence was estimated in southern Africa, where the expected difference in the probability of being on ART was 2.5%‐points (95% CrI: −3.8% to −1.1%) lower in urban areas. Conversely, in central and western Africa, the expected change in the probability of being on ART for woman living with HIV in urban areas was 6.5% (central Africa; 95% CrI: 3.5%, to 7.5%) and 5.0%‐points (western Africa; 95% CrI: 3.9−6.5%) higher compared to a similar woman living in a rural area.

In southern Africa, country‐level differences were the most influential predictors for both men and women, explaining 71% and 74% of the variation, respectively. The APCs for country differences were substantial, with expected changes of 7.9%‐points (95% CrI: 7.0−8.6%) for men and 8.1%‐points (95% CrI: 5.0−9.9%) for women.

Education and relative wealth impacted ART coverage in central and western Africa. The expected difference in the probability of being on ART between a woman with tertiary education and a similar woman with less than primary education was 5.7%‐points (95% CrI: 2.9−7.5%) in central Africa and 3.4%‐points (95% CrI: 2.3−3.8%) in western Africa.

Similarly, in western Africa, wealthier women had an expected 6.8%‐point higher probability of being on ART compared to poorer women (95% CrI: 4.0−7.8%).

District‐level variations accounted for as high as 22% of the total variance in ART coverage in western Africa. The expected difference in ART coverage between two otherwise identical individuals living in different districts of the same country was 13.9%‐points (95% CrI: 2.9−32.1%) for men and 10.3%‐points (95% CrI: 2.7−27.2%) for women in western Africa.

### 3.4 Sensitivity analyses

We performed sensitivity analyses by excluding either educational attainment or relative wealth from the models. The APC remained largely unchanged across these models, indicating that the exclusion of these variables did not significantly alter the estimated effects of other predictors (Figures S2.14−2.16).

## 4 DISCUSSION

We analysed surveys encompassing 2.3 million participants from 33 African countries over 20 years to investigate socio‐demographic and geographic inequalities in HIV prevalence, past‐year HIV testing and ART coverage. We found that educational attainment was associated with all outcomes—especially with HIV testing. Substantial within‐country variations in HIV outcomes also persisted, even when controlling for individual‐ and household‐level factors.

Individuals with tertiary education had a lower probability of living with HIV, were more likely to have recently tested for HIV and to be on ART (if living with HIV), compared to those with less than primary education. This pattern was especially clear for recent HIV testing uptake, but the strength of the relationship between education and HIV serostatus and ART coverage varied by region and sex. This differs from other studies from earlier periods in the epidemic that identified higher education as a risk factor for HIV acquisition [25, 40]. Our study incorporated multiple socio‐demographic factors and spatial variables at the district level, recognizing significant subnational variations in both HIV prevalence and educational attainment [12, 41].

This observed association between higher education and improved HIV outcomes could have important implications for future epidemic trajectories in Africa. Individuals with more formal schooling may be better able to navigate healthcare systems and more likely to adopt preventive behaviours. Collectively, these factors contribute to reduced population viraemia within more educated groups, thereby decreasing the potential for onward transmission. Given recent evidence of assortative mixing based on education [42], this dynamic may lead to divergent HIV incidence trends across educational strata, with less educated groups bearing a disproportionate share of new acquisitions. In the short and medium term, interventions aimed at mitigating these disparities could include targeted interventions that address barriers faced by people with fewer formal education, such as strengthening comprehensive sexuality education in schools to improve preventive behaviours [43], and deploying innovative learning tools to extend age‐ and sex‐appropriate prevention [44]. In the longer term, sustained investments in education—aligned with SDG 4—are crucial to build a more equitable foundation for HIV prevention and treatment access.

While education emerged as a strong predictor of HIV outcomes, the effect of relative wealth was not as consistent. Higher wealth was associated with greater recent HIV testing uptake and ART coverage among men in central Africa, but similar associations were not observed among women, possibly reflecting the equalizing effect of ANC services for women [39]. The association between wealth and HIV serostatus appeared to shift over time: earlier periods showed a higher probability of HIV among wealthier individuals, aligning with other studies [25, 45], but this association weakened or reversed in later periods.

We found higher HIV prevalence among older age groups, which is related to cumulative exposure and cohort effects during earlier decades when HIV incidence is higher [15]. Differences in access to testing and treatment were evident, with recent HIV testing more common among women, particularly women in the highest fertility age groups—likely associated with routine antenatal care services [39, 46]. Notably, in southern Africa, recent HIV testing was higher among adolescent girls and young women in recent years, potentially reflecting particular programmatic efforts to address HIV among this group [47, 48]. Conversely, ART coverage remained lower among younger age groups, especially men aged 25–34, possibly due to factors such as HIV stigma [49], which has been shown to lower engagement with healthcare services [50, 51, 52].

We observed substantial subnational variations in HIV prevalence and ART coverage among PLHIV, even when considering individual‐ and household‐level factors, highlighting the localized nature of HIV epidemics [12, 13]. District‐level differences in the implementation of HIV responses can lead to disparities in service availability, emphasizing the need for geographically tailored interventions and equitable resource distribution [53, 54].

Several limitations should be acknowledged. First, our study did not address certain determinants such as sexual behaviours and identity, sex work, and gender identity, which can be important drivers of HIV burden and barriers to care but are currently not adequately captured in nationally representative household surveys [55, 56, 57, 58]. Second, the wealth index used has limitations as a measure of SES, particularly in multi‐country analyses [59]. We mitigated this by calculating relative wealth quintiles within place of residence for each survey, implicitly embedding an interaction between wealth and place of residence. This ensures that our metric for wealth has a consistent interpretation across settings [60], but limits our ability to add further interaction terms—for instance, between wealth and education. Further research is needed to develop more robust indicators of SES and explore their interaction with education [61]. Third, we used Kish normalization to calculate effective sample sizes, which accounts for heterogeneous sampling weights but not clustered sampling design. Fourth, although we compared multiple models to identify the best fit for each outcome, the optimal model varied across outcomes, regions and sexes. This cannot solely be attributed to differences in the relative importance of model components; it also reflects the heterogeneity in data availability and quality in different settings and for different outcomes. Finally, as our study is based on observational data and employs predictive modelling, the associations observed should not be interpreted as causal relationships. Although removing education or wealth in sensitivity analyses produced only minor shifts in the posterior estimates of the remaining covariates, this does not rule out confounding by unmeasured dimensions of SES, such as occupation or social capital.

Our findings have important implications for HIV policy and programming. The strong associations between education and HIV outcomes underscore the risk of a growing socio‐demographic gradient in HIV incidence due to the correlation of higher HIV prevalence and lower ART coverage among people with less formal education. While enhancing educational opportunities remains a crucial long‐term strategy—aligned with SDG 4—actionable steps can be taken now to target the needs of individuals with less formal education and narrow the observed gaps in HIV prevention and treatment.

## 5 CONCLUSIONS

Our comprehensive analysis underscores significant socio‐demographic and geographic inequalities in HIV outcomes across Africa, with educational attainment emerging as a crucial determinant of recent HIV testing, and an important—though context‐dependent—factor for both HIV prevalence and treatment coverage. By systematically collating data from multiple countries and time periods, our findings clarify previously mixed evidence on socio‐economic proxies, indicating a robust and consistent protective effect of education. Addressing educational disparities through targeted, culturally competent interventions and equitable resource allocation is essential to progress towards universal quality schooling, which is indispensable for sustaining gains in HIV prevention and treatment.

## COMPETING INTERESTS

JWI‐E's institution receives funding from the Gates Foundation, UNAIDS, and the US National Institutes of Health, and has received consulting fees from BAO Systems and support to attend meetings from UNAIDS, the Gates Foundation, the International AIDS Society, and the South African Centre for Epidemiological Modelling and Analysis.

JWI‐E is a Deputy Editor of the Journal of the International AIDS Society.

## AUTHORS’ CONTRIBUTIONS

AA, MM‐G and JWI‐E developed the research project. AA, SK, JS and YX prepared and reviewed data inputs. AA developed the statistical models. All authors contributed to the interpretation of the study results. AA wrote the first draft of the manuscript. All authors critically edited the manuscript for intellectual content and approved the final version of the manuscript for publication.

## FUNDING

Some of this work was supported by UNAIDS (agreement for performance of work 2022/1277648), and this research was supported by the Canadian Institute of Health Research.

AA was supported through a Fonds de la recherche du Québec—Santé (FRQS) *Post‐Doctoral Fellowship*. MM‐G's research programme is funded by a Canada Research Chair (Tier 2) in Population Health Modeling. JWI‐E was supported by UNAIDS, the Bill & Melinda Gates Foundation (grant number INV‐006733), the National Institute of Allergy and Infectious Diseases of the US National Institutes of Health (award number R01AI136664) and the Medical Research Council Centre for Global Infectious Disease Analysis (reference MR/R015600/1), which was jointly funded by the UK Medical Research Council and the UK Foreign, Commonwealth and Development Office (under the Medical Research Council and the UK Foreign, Commonwealth and Development Office Concordat agreement) and is also part of the European and Developing Countries Clinical Trials Partnership programme supported by the EU.

For the purpose of open access, the author(s) has applied a Creative Commons Attribution (CC BY) licence (where permitted by UKRI, “Open Government Licence” or “Creative Commons Attribution No‐derivatives (CC BY‐ND) licence” may be stated instead) to any Author Accepted Manuscript version arising.

Code used for statistical analyses and modelling is available from GitHub: https://github.com/aallorant/district‐and‐demographic‐trends‐hiv.

## Supporting information




**Figure S1.1**: Distribution of the proportion of households in each urban/rural strata and five wealth quintiles, across 103 population‐based surveys (DHS, AIS, PHIA, BAIS, SABSSM) conducted between 2003 and 2022 in sub‐Saharan Africa.
**Table S1.1**: Models considered to estimate the three outcomes.
**Table S2.1**: Data availability of population‐based survey data collecting information on recent HIV testing, HIV prevalence and ART coverage data by region of sub‐Saharan Africa.
**Table S2.2**: Socio‐demographic characteristics for recent HIV testing sample.
**Table S2.3**: Socio‐demographic characteristics for HIV prevalence sample.
**Table S2.4**: Socio‐demographic characteristics for ART coverage sample.
**Figure S2.1**: Dominant variance components in multilevel models of HIV outcomes by region and sex.
**Figure S2.2**: Expected difference in the probability of living with HIV among all men and women, in sub‐Saharan Africa given a change in the covariates.
**Figure S2.3**: Expected difference in the probability of recent HIV testing among all men and women, in sub‐Saharan Africa, given a change in the covariates.
**Figure S2.4**: Expected difference in the probability of ART coverage among all men and women living with HIV, in sub‐Saharan Africa, given a change in the covariates.
**Figure S2.5**: Odds ratio of living with HIV, by factor, time period and sex.
**Figure S2.6**: Odds ratio of recent HIV testing, by factor, time period and sex.
**Figure S2.7**: Odds ratio of ART coverage among PLHIV, by factor, time period and sex.
**Figure S2.8**: Performance for the logistic regression model of HIV status among women, for the five model formulations, according to CPO, DIC and WAIC.
**Figure S2.9**: Performance for the logistic regression model of HIV status among men, for the five model formulations, according to CPO, DIC and WAIC.
**Figure S2.10**: Performance for the logistic regression model of recent HIV testing among women, for the five model formulations, according to CPO, DIC and WAIC.
**Figure S2.11**: Performance for the logistic regression model of recent HIV testing among men, for the five model formulations, according to CPO, DIC and WAIC.
**Figure S2.12**: Performance for the logistic regression model of ART coverage among women living with HIV, for the five model formulations, according to CPO, DIC and WAIC.
**Figure S2.13**: Performance for the logistic regression model of ART coverage among men living with HIV, for the five model formulations, according to CPO, DIC and WAIC.
**Figure S2.14**: Differences in Average Predictive Comparisons for HIV status between main model and sensitivity analyses.
**Figure S2.15**: Differences in Average Predictive Comparisons for recent HIV testing between main model and sensitivity analyses.
**Figure S2.16**: Differences in Average Predictive Comparisons for ART coverage among people living with HIV between main model and sensitivity analyses.

## Data Availability

DHS and PHIA household survey data are publicly available upon registration and request from https://dhsprogram.com/ and PHIA https://phia‐data.icap.columbia.edu/datasets/. Administrative boundaries were retrieved from the Global Administrative Unit Layers dataset.
