# Supplementary material for: Socio‐demographic and geographic disparities in HIV prevalence, HIV testing and treatment coverage: An analysis of 108 national household surveys in 33 African countries
Source: J Int AIDS Soc. 2025 Aug 13;28(8):e70024. doi: 10.1002/jia2.70024 (PMC12350184; doi:10.1002/jia2.70024)
Supplement: Supplementary file 1 — Figure S1.1: Distribution of the proportion of households in each urban/rural strata and five wealth quintiles, across 103 population‐based surveys (DHS, AIS, PHIA, BAIS, SABSSM) conducted between 2003 and 2022 in sub‐Saharan Africa. Table S1.1: Models considered to estimate the three outcomes. Table S2.1: Data availability of population‐based survey data collecting information on recent HIV testing, HIV prevalence and ART coverage data by region of sub‐Saharan Africa. Table S2.2: Socio‐demographic characteristics for recent HIV testing sample. Table S2.3: Socio‐demographic characteristics for HIV prevalence sample. Table S2.4: Socio‐demographic characteristics for ART coverage sample. Figure S2.1: Dominant variance components in multilevel models of HIV outcomes by region and sex. Figure S2.2: Expected difference in the probability of living with HIV among all men and women, in sub‐Saharan Africa given a change in the covariates. Figure S2.3: Expected difference in the probability of recent HIV testing among all men and women, in sub‐Saharan Africa, given a change in the covariates. Figure S2.4: Expected difference in the probability of ART coverage among all men and women living with HIV, in sub‐Saharan Africa, given a change in the covariates. Figure S2.5: Odds ratio of living with HIV, by factor, time period and sex. Figure S2.6: Odds ratio of recent HIV testing, by factor, time period and sex. Figure S2.7: Odds ratio of ART coverage among PLHIV, by factor, time period and sex. Figure S2.8: Performance for the logistic regression model of HIV status among women, for the five model formulations, according to CPO, DIC and WAIC. Figure S2.9: Performance for the logistic regression model of HIV status among men, for the five model formulations, according to CPO, DIC and WAIC. Figure S2.10: Performance for the logistic regression model of recent HIV testing among women, for the five model formulations, according to CPO, DIC and WAIC. Figure S2.11: Performance for the [file JIA2-28-e70024-s001.pdf]

Socio-Demographic and Geographic Disparities in HIV Prevalence, HIV testing, and Treatment Coverage: An Analysis of 108 National Household Surveys in 33 African Countries

Adrien Allorant, Salome Kuchukhidze, James Stannah, Yiqing Xia, Sanele S Masuku, Gatien K Ekanmian, Jeffrey W Imai-Eaton, Mathieu Maheu-Giroux

Correspondence to: Adrien Allorant (adrien.allorant@soton.ac.uk)

Table of Contents

1. Supplementary Methods 2
2. Supplementary Tables and Figures 9

1. Supplementary Methods

Survey-weighted likelihoods

To account for the complex design of the household surveys used in this analysis, we replaced observed counts y^l (with *l* either recent HIV testing, HIV positive test, or ART among PLHIV) and sample sizes m^l with effective counts $\tilde{y}^l$ and sample sizes $\tilde{m}^l$, by normalizing adequate survey weights – sampling weights for recent HIV testing analysis, and HIV sampling weights for HIV prevalence, and ART coverage among PLHIV analyses. Specifically, following Thomas *et al*. (60), we normalized each individual *i* in survey *s* residing in strata *j* (defined by the survey region and urban/rural geographic strata) sampling weight ω_{ijs} using the Kish effective sample size:

$$\tilde{\omega}_{ijs} = \frac{\omega_{ijs}}{\overline{\omega_{Js}}} \frac{M_s}{M_{eff}}$$

Where $\overline{\omega_{Js}}$ is calculated as the arithmetic mean of the sampling weights of individuals in strata *j* and survey *s*, M_s corresponds to the survey *s* sample size, and M_{eff} is the Kish effective sample size, which accounts for heterogeneity in sampling weights between survey strata, and is calculated as:

$$M_{eff} = \frac{\left(\sum_i \omega_{ijs}\right)^2}{\sum_i \omega_{ijs}^2}$$

Model fits

We modelled the survey-weighted counts of survey respondents with a recent HIV test or a positive HIV test, and the number of people living with HIV on ART, with socio-demographic characteristics *k,* sampled in area *d*, and year *t*, using a Binomial likelihood:

$$\tilde{y}^l_{kdt} \sim \text{Binomial}(\tilde{m}^l_{kdt}, p^l_{kdt})$$

Where the logit of the probability is modelled as:

$$\text{logit}\left(p^l_{kdt}\right) = X_k^T\beta + u_d + w_c + v_t + \phi_{tc} + \psi_{td} + X_{kp}^T\gamma_p$$

Here:

- $X_k^T\beta$ represents the fixed effects of socio-demographic characteristics (e.g., age, place of residence, education, relative wealth).
- u_d is the random effects for the subnational area d.
- v_t is the random effect for year t, modeled with an AR1 process.
- w_c is the random effect for the country c.
- ψ_{td} represents the district-year interaction effects, allowing for different temporal trends by district.
- ϕ_{tc} represents the country-year interaction effects, allowing for different temporal trends by country.

- $X_{kp}^{T}\gamma_p$ includes the random slopes by period p (2003-2007, 2008-2012, 2013-2017, 2018-2023) for the socio-demographic covariates (e.g., age, place of residence, education, relative wealth).

The model components in black are included in all five models, while the terms in blue are only included for some versions of the model.

Prior distribution for fixed effects of socio-demographic characteristics:

$$\beta_j \sim N\left(0, \tau_{\beta-1}\right), \tau_\beta = 0.001$$

Random effect for subnational area:

$$\mathrm{u_d} \sim \mathrm{N}(0, \sigma_\mathrm{u}^2) \quad \text{(iid RE) or } \mathrm{u_d} \sim \mathrm{ICAR}(\mathrm{Q_u}, \sigma_\mathrm{u}^2)$$

with a **precision hyper-prior:** $\tau_u = \sigma_u^{-2} \sim PC(P(\sigma_u > 2.5) = 0.01)$

AR1 random effect for year:

$$\mathrm{v_t} \sim \mathrm{AR1}$$
$$\mathrm{v_t} = \rho \mathrm{v_{t-1}} + \eta_\mathrm{t}$$
$$\text{where } \eta_\mathrm{t} \sim \mathrm{N}(0, \sigma_\mathrm{v}^2) \ |\rho| < 1$$

with a **precision hyper-prior:** $\tau_v = \sigma_v^{-2} \sim PC(P(\sigma_v > 2.5) = 0.01)$

IID random effect for all other random effects:

$$\mathrm{w_c} \sim \mathrm{N}(0, \sigma_\mathrm{w}^2)$$

with a **precision hyper-prior:** $\tau_c = \sigma_c^{-2} \sim PC(P(\sigma_c > 2.5) = 0.01)$

Relative Wealth Quintiles

With the original DHS wealth quintiles, rural households are predominantly classified into the poorest (Q1 and Q2) while urban households are largely placed in the richer (Q4 and Q5) quintiles (Figure S1). This is likely due to the DHS wealth index tendency to be 'pro-urban', by including certain assets (e.g., running water, electricity) that can be publicly provided in urban areas, therefore misclassifying urban poor households are richer than they really are. The DHS quintiles obscure this reality by clustering nearly all urban households into the higher quintiles, thereby masking the presence of economically disadvantaged groups within urban settings. The exact impact of geographic strata on wealth distribution necessarily varies across different countries. This inconsistency makes cross-country comparisons using DHS quintiles unreliable, as the definition of each quintile can differ based on the country-specific wealth distribution within strata.

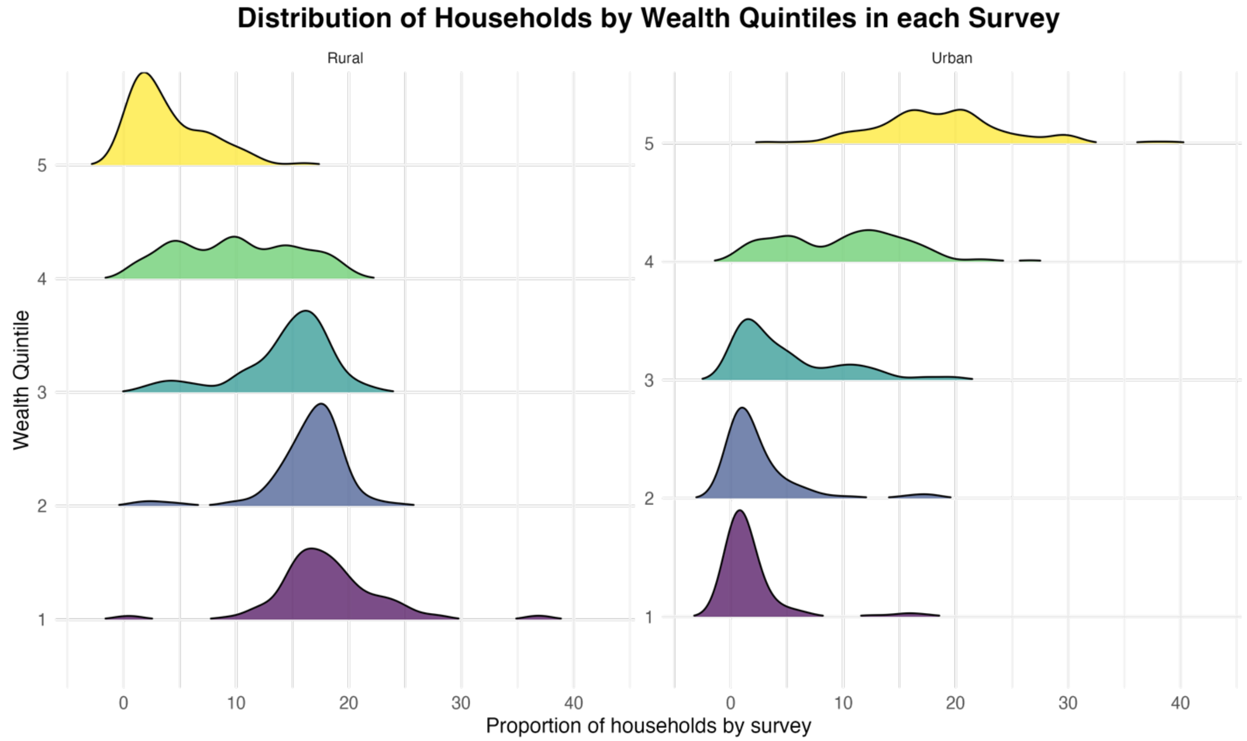

Figure S1.1. Distribution of the proportion of households in each urban/rural strata and five wealth quintiles, across 103 population-based surveys (DHS, AIS, PHIA, BAIS, SABSSM) conducted between 2003 and 2022 in sub-Saharan Africa. Across surveys, most rural households tend to be classified in the lowest wealth quintiles, while urban households tend to be classified in richer quintiles. This illustrates the 'pro-urban' bias of the original wealth quintile. WIQ= Wealth Index Quintiles, Q1 = Poorest 20% households, Q5 = Richest 20% households. AIS: AIDS Indicator Survey; DHS: Demographic and Health Surveys; PHIA: Population-based HIV Impact Assessment; MICS: Multiple Indicator Cluster Surveys.

In the absence of more robust indicators of socio-economic status, calculating a relative wealth index addresses some of these challenges:

1. By ranking households within their strata (e.g., urban vs. rural), the relative wealth index ensures that socio-economic variations within each geographic category are accurately captured. This approach recognizes that social stratification exists in both urban and rural areas.
2. Relative wealth quintiles enable consistent comparisons within similar environments across different regions and countries. For instance, a household in the poorest quintile within an urban stratum can be directly compared to a household in the poorest quintile within a rural stratum, both representing the lowest socio-economic status within their respective environments.

Average Predictive Comparisons

We employed Average Predictive Comparisons (APCs) to quantify the associations between covariates and the outcomes of interest in our models. APCs measure the expected change in the probability of the outcome when changing a covariate from one category to another, holding all other covariates constant at their observed values. This approach provides effect estimates directly interpretable on the outcome scale, which is particularly valuable in models with interaction terms where odds ratios may not be easily interpretable. Additionally, APCs can be used to summarize the importance of spatial random effects, although in that case their interpretation differs from APCs for socio-demographic variables; they are a measure of absolute magnitude, as districts and countries are unordered variables. APCs for districts (respectively countries) measure the expected difference in probability of an outcome for two individuals sharing the same socio-demographic characteristics, but who live in two different districts of the same country (respectively, two different countries of the same region).

Calculation of APCs for Socio-Demographic Covariates
Individual Predicted Probabilities
For a categorical covariate X_k with categories $l = 1, \ldots, L$, we calculate the predicted probabilities under two scenarios:

1. **Reference Category (l_0)**:

$$p_i^{l_0} = \frac{1}{1 + \exp\left(-\left(\beta_0 + \beta_k^{l_0} + \sum_{\{j \neq k\}} \beta_j X_{ij} + \sum_m \theta_m Z_{im}\right)\right)}$$

Where $\beta_k^{l_0}$ is the coefficient for the reference category of covariate X_k.

2. **Target Category (lll)**:

$$p_i^{l} = \frac{1}{1 + \exp\left(-\left(\beta_0 + \beta_k^{l} + \sum_{\{j \neq k\}} \beta_j X_{ij} + \sum_m \theta_m Z_{im}\right)\right)}$$

Where β_k^{l} is the coefficient for category *l* of covariate X_k.

Calculating the Difference in Probabilities
For each individual i, the difference in predicted probabilities when changing X_k from the reference category to category *l* is:

$$\Delta\, p_i(l)^{(s)} = {p_i^{(s)}}_l - {p_i^{(s)}}_{l_0}$$

Averaging Over Individuals
The APC for covariate X_k from the reference category to category *l* is calculated by averaging over all individuals:

$$\overline{\Delta \mathrm{p}\ (l)^{(s)}} = \frac{1}{N}\sum_{i=1}^{N} \Delta\, \mathrm{p_i}(l)^{(s)}$$

The mean APC is obtained by averaging all samples s = 1, …, 1,000:

$$\overline{\Delta\, \mathrm{p(l)}} = \frac{1}{1000}\sum_{i=1}^{1000} \overline{\Delta \mathrm{p}\ (l)^{(s)}}$$

We also computed the 2.5th and 97.5th percentiles of the posterior distribution of $\overline{\Delta \mathrm{p}\ (l)^{(s)}}$ to obtain a 95% credible interval.

Assessment of the Impact of Including Both Education and Relative Wealth in the Model

To assess multicollinearity, we calculated Spearman's rank correlation coefficient ρ between education and wealth across all surveys.

To evaluate how the inclusion or exclusion of education and relative wealth affects the estimates of each other in our models, we also conducted sensitivity analyses. Specifically, we re-ran our primary models under different specifications and compared the posterior estimates of education and wealth.

We considered three model specifications:

1. **Main Model (Model A)**: Includes both education and relative wealth as predictors.
2. **Sensitivity Model without Wealth (Model B)**: Includes education but excludes relative wealth.
3. **Sensitivity Model without Education (Model C)**: Includes relative wealth but excludes education.

Posterior Sample Extraction

To assess the impact of excluding education or wealth on the estimated effects of other variables, we compared the Average Predictive Comparisons (APCs) between the **Main** model and the

sensitivity analyses (**No Education** and **No Wealth** models). The variables of interest included education, relative wealth, place of residence, and age group.
For each model (Main, No Education, No Wealth), we extracted posterior samples of the APCs for each category of these variables. The APCs represent the expected change in the probability of the outcome when transitioning from the baseline category to a specific category of a variable, holding all other variables constant.

To quantify the effect of excluding a variable, we calculated the sample-to-sample differences in APCs between the Main model and each sensitivity model. Specifically, for each posterior sample $s = 1, \dots, 1000$, we computed:

- **Between Main Model (A) and No Wealth Model (B):**

$$\Delta\, \vartheta_{\text{var}}{}^{(s)} = \vartheta_{\text{var}}{}^{(s)}{}_{A} - \vartheta_{\text{var}}{}^{(s)}{}_{B}$$

- **Between Main Model (A) and No Wealth Model (C):**

$$\Delta\, \vartheta_{\text{var}}{}^{(s)} = \vartheta_{\text{var}}{}^{(s)}{}_{A} - \vartheta_{\text{var}}{}^{(s)}{}_{C}$$

Here, $\vartheta_{\text{var}}{}^{(s)}$ denotes the APC for a specific variable (education, wealth, place of residence, or age group) at sample s. This computation was performed for each category of the variables, compared to their baseline category.

For each difference $\Delta\vartheta_{\text{var}}{}^{(s)}$, we computed:

$$\overline{\Delta\, \vartheta_{\text{var}}} = \frac{1}{1000} \sum_{s=1}^{1000} \Delta\vartheta_{\text{var}}{}^{(s)}$$

We also computed the 2.5th and 97.5th percentiles of the posterior distribution of $\Delta\, \vartheta_{\text{var}}{}^{(s)}$ to obtain a 95% credible interval.

By analyzing the differences $\Delta\vartheta_{\text{var}}{}^{(s)}$, we assessed whether excluding education or wealth from the models significantly altered the estimated effects of the other variables. If the credible intervals of the differences included zero and the mean differences were negligible, it indicated that the exclusion did not substantially impact the APCs of the other variables.

Posterior distributions of all model parameters and hyperparameters were estimated using the integrated nested Laplace approximation (INLA) (39) implemented with the statistical package R-INLA version 22.12.16 (40) in R version 4.4.1. Central estimates and 95% credible intervals (CrI)

were obtained by drawing 1,000 posterior samples for all parameters estimated in the model and calculating the mean, 2.5th, and 97.5th percentiles. R code reproducing the analysis are available from https://github.com/aallorant/district-and-demographic-trends-hiv.

Model selection

For each indicator, we compared the 5 models presented in table SM1.1 using three selection procedures (the deviance information criteria [3], the Watanabe-Akaike information criteria [4], and the sum of log-conditional predictive ordinate [5]). In the absence of consensus on a single criterion, when different criteria pointed to different models, we used a majority rule. Model selection results for each of the three outcomes are presented in separate Figures- Figures S2.8-S2.13.

Model	Formula
1	$X_k^T\beta + u_d + w_c + v_t$
2	$X_k^T\beta + u_d + w_c + v_t + \phi_{tc} + \psi_{td}$ where $u_d \sim N(0, \sigma_u^2)$
3	$X_k^T\beta + u_d + w_c + v_t + \phi_{tc} + \psi_{td} + X_{kp}^T\gamma_p$ where $u_d \sim N(0, \sigma_u^2)$
4	$X_k^T\beta + u_d + w_c + v_t + \phi_{tc} + \psi_{td}$ where $u_d \sim \mathrm{ICAR}(\mathrm{Q_u}, \sigma_u^2)$
5	$X_k^T\beta + u_d + w_c + v_t + \phi_{tc} + \psi_{td} + X_{kp}^T\gamma_p$ where $u_d \sim \mathrm{ICAR}(\mathrm{Q_u}, \sigma_u^2)$

Table S1.1: Models considered to estimate the 3 outcomes. Models 2 and 3 use independent and identically distributed random effects, while Models 4 and 5 use intrinsic conditional auto-regressive terms (ICAR).

2. Supplementary Tables and Figures

Supplementary Data

Table S2.1: Data availability of population-based survey data collecting information on recent HIV testing, HIV prevalence, and ART coverage data by region of sub-Saharan Africa.

Region	Outcome	Effective Sample Size (N)	Number of surveys (N)
**Sub-Saharan Africa**	Recent HIV testing	2,736,943	108
	HIV status	1,700,020	83
	ART coverage	42,203	22
**Central Africa**	Recent HIV testing	312,102	14
	HIV status	193,383	11
	ART coverage	623	1
**Eastern Africa**	Recent HIV testing	1,210,099	49
	HIV status	767,866	36
	ART coverage	20,238	12
**Southern Africa**	Recent HIV testing	331,346	17
	HIV status	266,566	16
	ART coverage	19,471	7
**Western Africa**	Recent HIV testing	883,396	29
	HIV status	472,205	20
	ART coverage	1,871	2

Table S2.2: Sociodemographic characteristics for Recent HIV testing sample

Variable	Category	Effective Sample Size (N)	Percentage
Sex	Male	841,183	36.2
Sex	Female	1,484,885	63.8
Age group	15-19	850,423	36.6
Age group	25-29	691,525	29.7
Age group	35-39	646,631	27.8
Age group	50-54	137,489	5.9
Residence	Rural	1,392,395	59.9
Residence	Urban	933,673	40.1
Education	No education	523,969	22.5
Education	Primary	817,177	35.1
Education	Secondary	762,407	32.8
Education	Higher	158,061	6.8
Relative Wealth quintile	Poorest	419,443	18.0
Relative Wealth quintile	Poorer	430,659	18.5
Relative Wealth quintile	Middle	432,603	18.6
Relative Wealth quintile	Richer	441,734	19.0
Relative Wealth quintile	Richest	454,378	19.5
Total		2,326,068	100.0

Table S2.3: Sociodemographic characteristics for HIV prevalence sample

Variable	Category	Effective Sample Size (N)	Percentage
Sex	Male	815,567	48.2
Sex	Female	874,762	51.8
Age group	15-19	623,794	36.9
Age group	25-29	463,473	27.4
Age group	35-39	437,040	25.9
Age group	50-54	166,022	9.8
Residence	Rural	999,761	59.1
Residence	Urban	690,567	40.9
Education	No education	313,466	18.5
Education	Primary	587,542	34.8
Education	Secondary	588,550	34.8
Education	Higher	122,410	7.2
Relative Wealth quintile	Poorest	310,848	18.4
Relative Wealth quintile	Poorer	313,678	18.6
Relative Wealth quintile	Middle	311,937	18.5
Relative Wealth quintile	Richer	313,849	18.6
Relative Wealth quintile	Richest	317,531	18.8
Total		1,690,329	100.0

Table S2.4: Sociodemographic characteristics for ART coverage sample

Variable	Category	Effective Sample Size (N)	Percentage
Sex	Male	19,751	36.0
Sex	Female	35,103	64.0
Age group	15-19	5,663	10.3
Age group	25-29	14,511	26.5
Age group	35-39	23,931	43.6
Age group	50-54	10,748	19.6
Residence	Rural	31,387	57.2
Residence	Urban	23,466	42.8
Education	No education	5,745	10.5
Education	Primary	24,216	44.1
Education	Secondary	20,449	37.3
Education	Higher	3,831	7.0
Relative Wealth quintile	Poorest	10,795	19.7
Relative Wealth quintile	Poorer	10,515	19.2
Relative Wealth quintile	Middle	10,740	19.6
Relative Wealth quintile	Richer	10,772	19.6
Relative Wealth quintile	Richest	9,824	17.9
Total		54,853	100.0

Supplementary Figures

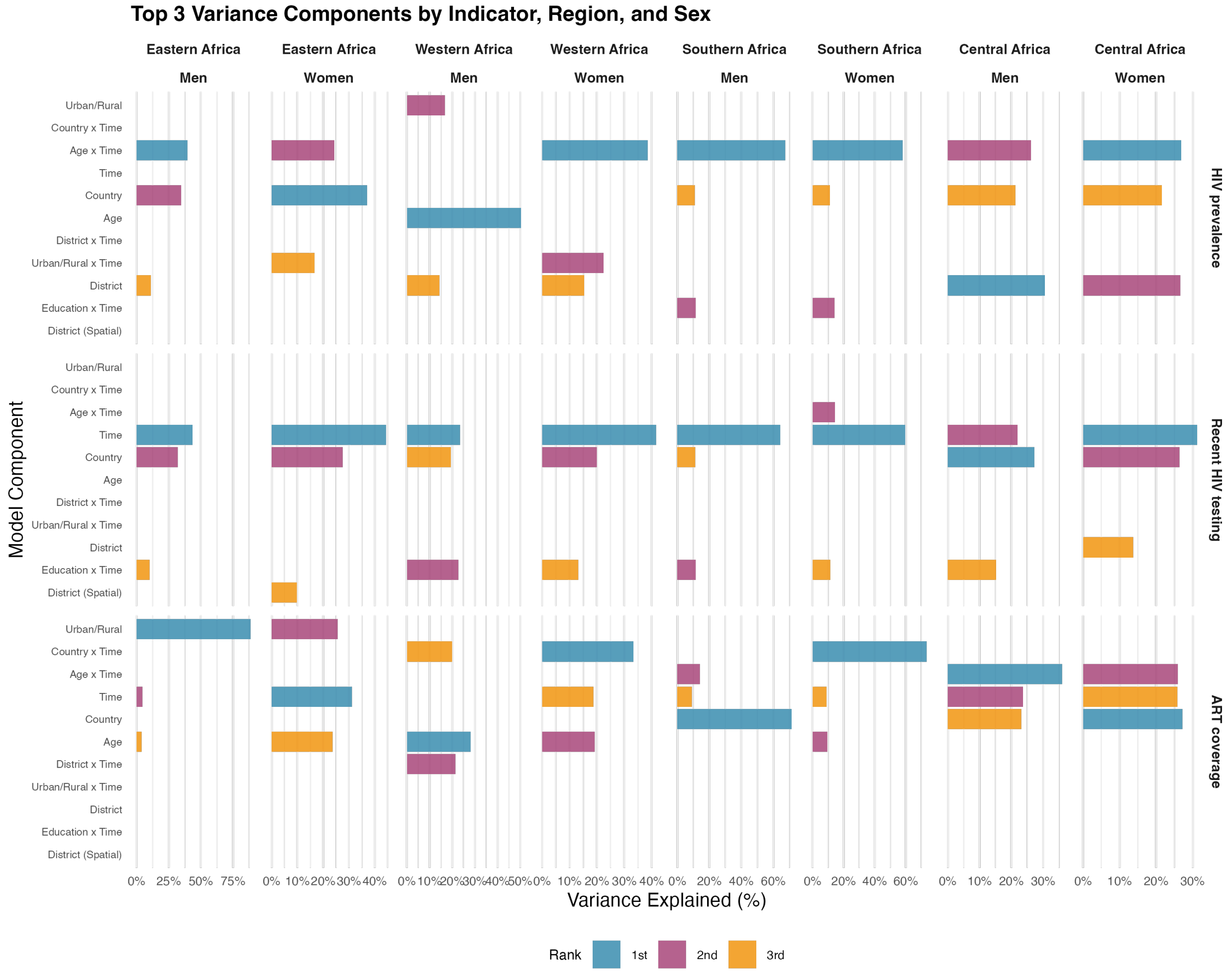

Figure S2.1: Dominant variance components in multilevel models of HIV outcomes by region and sex. Three variance components explaining the largest percentage of variation from best multilevel logistic regression models for HIV prevalence, recent HIV testing, and ART coverage across four regions. Colors indicate ranking: first (blue), second (pink), third (orange) largest contributors to model variance.

Average Predictive Comparisons stratified by period

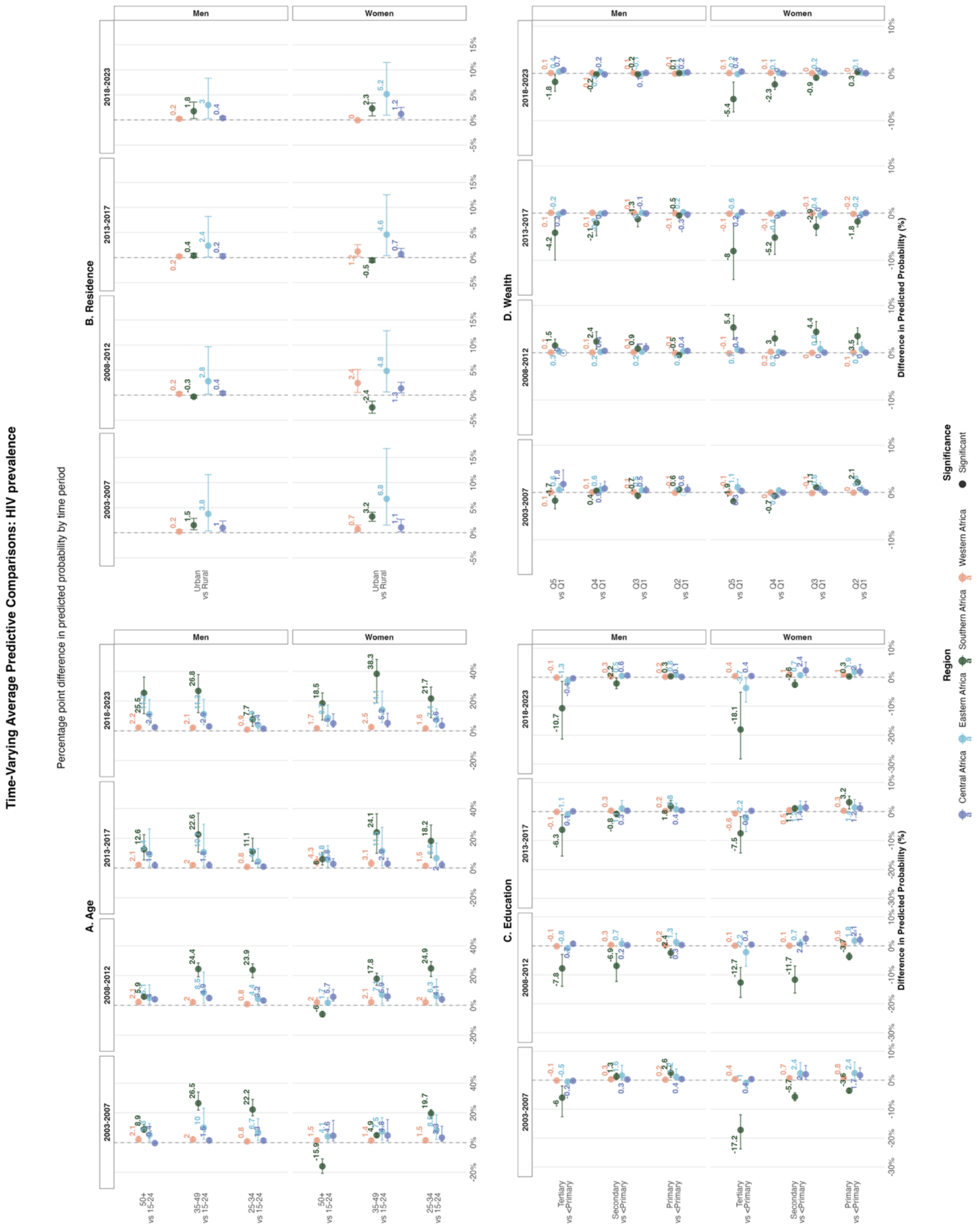

Figure S2.2: Expected difference in the probability of living with HIV among all men and women, in sub-Saharan Africa given a change in the covariates. Average predictive comparisons for the four socio-demographic covariates, age (A), place of residence (B), education

(C), and relative wealth quintile (D). Average predictive comparisons represent estimates of the expected difference in the probability of living with HIV between two respondents with different values for one covariate but similar values for all others, obtained by post-estimation simulation from the model. The mean effect estimates are displayed above the circles. Horizontal lines are 95% credible intervals. Empty circles indicate estimates whose credible intervals include zero. The model was stratified by regions of sub-Saharan Africa. Effect estimates for each region are presented in different colors.

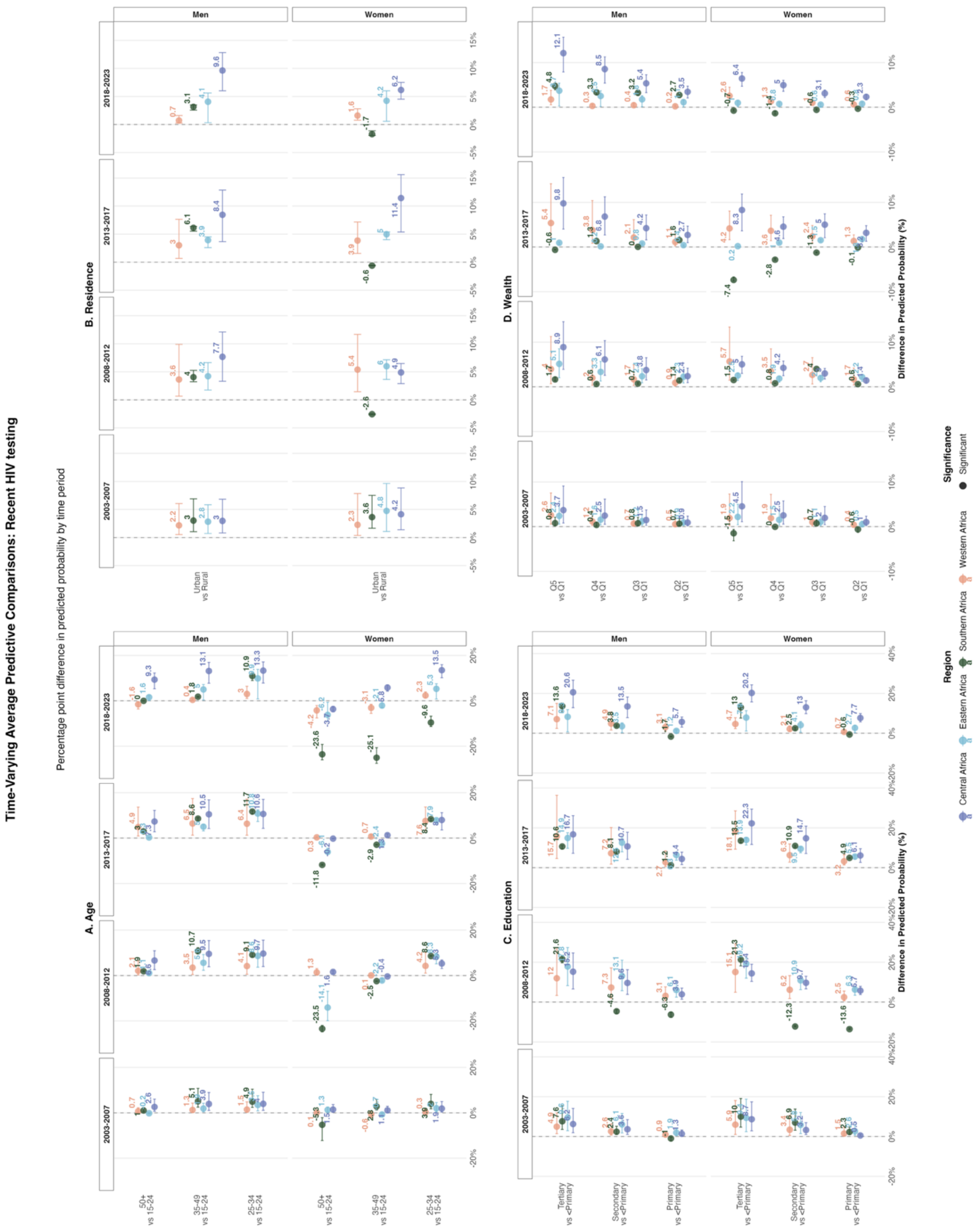

Figure S2.3: Expected difference in the probability of recent HIV testing among all men and women, in sub-Saharan Africa given a change in the covariates. Average predictive comparisons for the four socio-demographic covariates, age (A), place of residence (B), education (C), and relative wealth quintile (D). Average predictive comparisons represent estimates of the

expected difference in the probability of recent HIV testing between two respondents with different values for one covariate but similar values for all others, obtained by post-estimation simulation from the model. The mean effect estimates are displayed above the circles. Horizontal lines are 95% credible intervals. Empty circles indicate estimates whose credible intervals include zero. The model was stratified by regions of sub-Saharan Africa. Effect estimates for each region are presented in different colors.

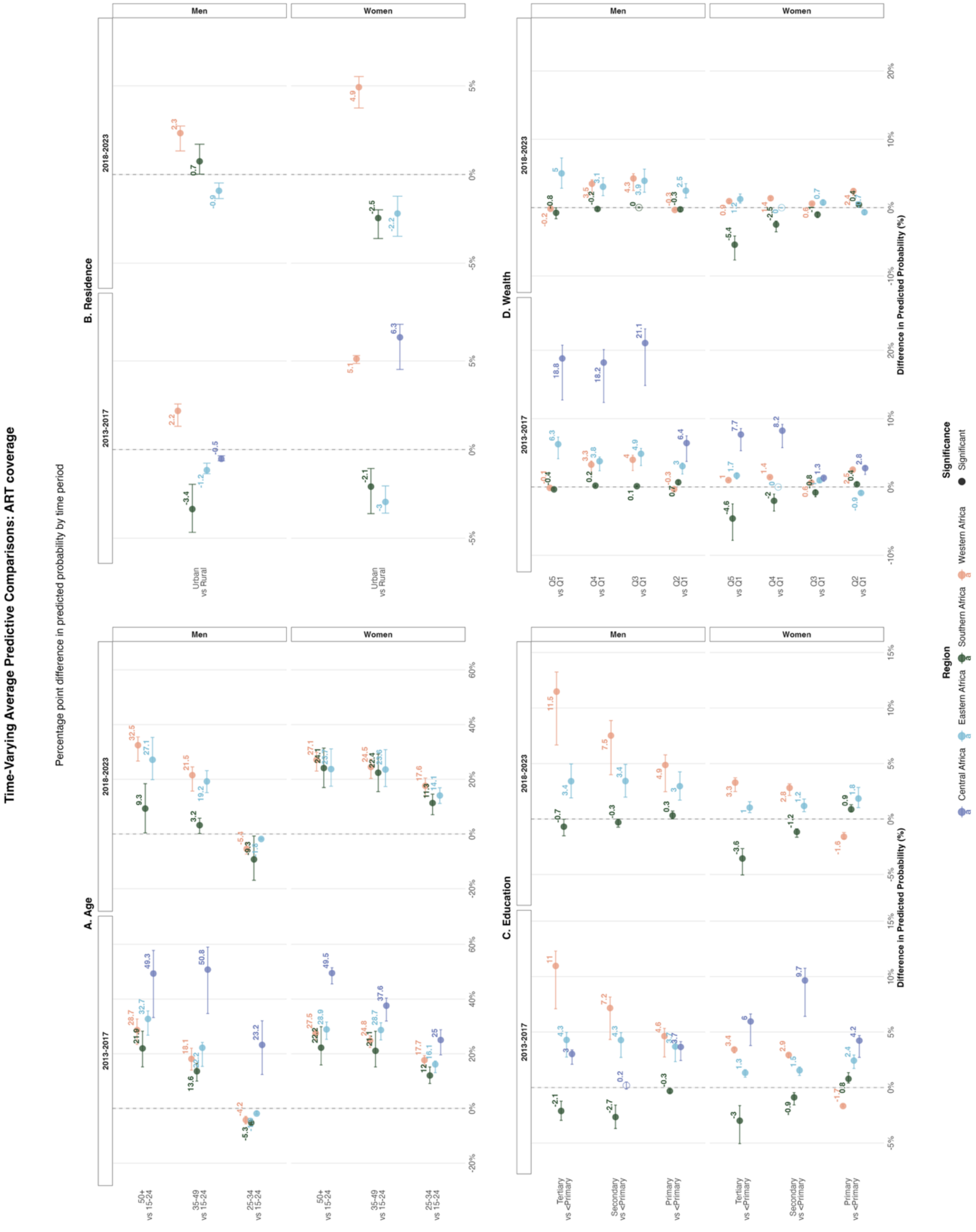

Figure S2.4: Expected difference in the probability of ART coverage among all men and women living with HIV, in sub-Saharan Africa given a change in the covariates. Average predictive comparisons for the four socio-demographic covariates, age (A), place of residence (B), education (C), and relative wealth quintile (D). Average predictive comparisons represent

estimates of the expected difference in the probability of ART coverage between two respondents living with HIV with different values for one covariate but similar values for all others, obtained by post-estimation simulation from the model. The mean effect estimates are displayed above the circles. Horizontal lines are 95% credible intervals. Empty circles indicate estimates whose credible intervals include zero. The model was stratified by regions of sub-Saharan Africa. Effect estimates for each region are presented in different colors.

Estimated Odds-Ratios

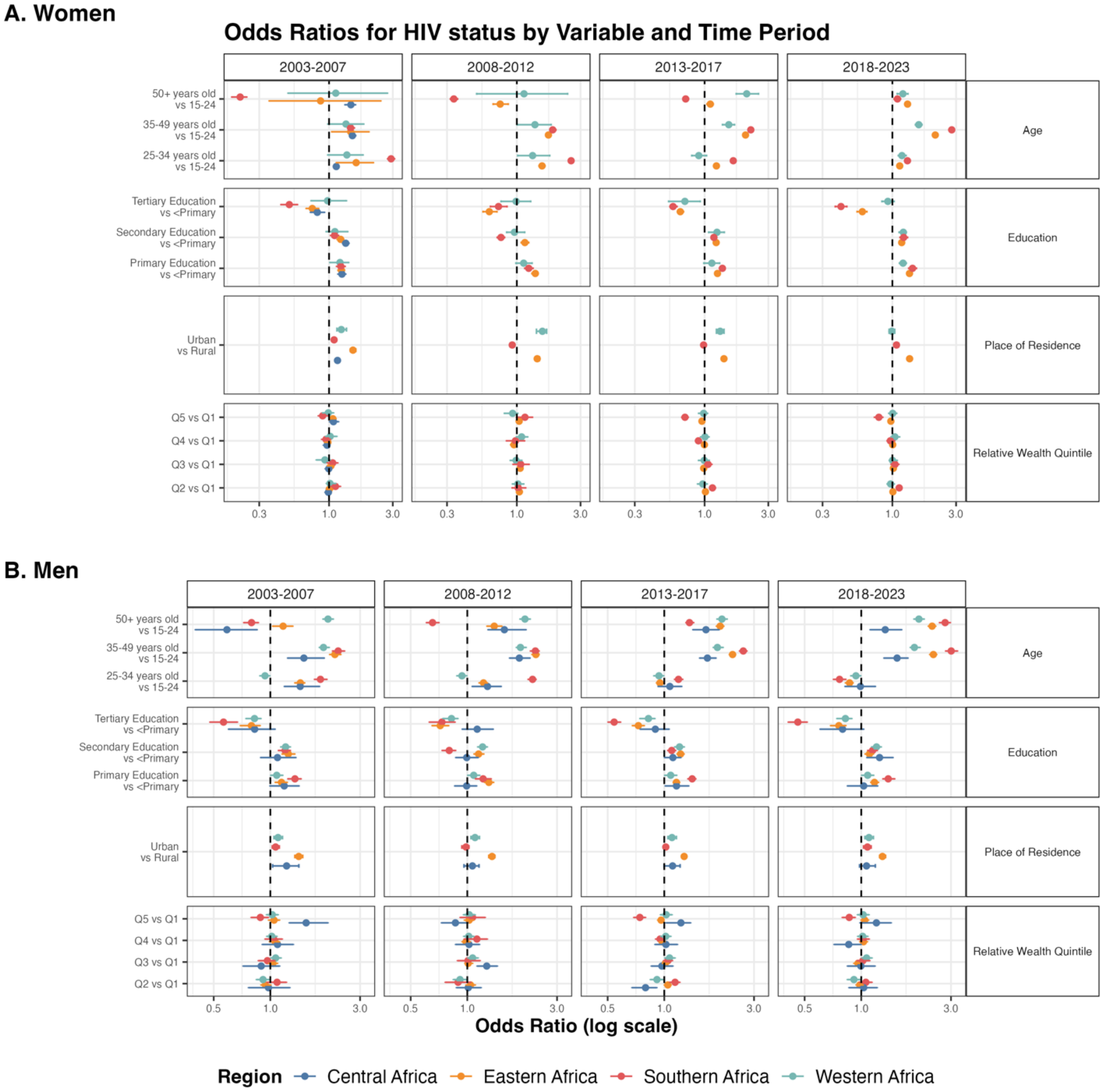

Figure S2.5: Odds-ratio of living with HIV, by factor, time-period, and sex. This figure shows the estimated odds-ratio derived from a hierarchical regression model of HIV seroprevalence, for the four socio-demographic covariates, age, place of residence, education and relative wealth quintile, among women (A), and men (B). The mean effect estimates are depicted as circles. Horizontal lines are 95% credible intervals. The model was stratified by sex, and regions of sub-Saharan Africa. Effect estimates for each region are presented in different colors.

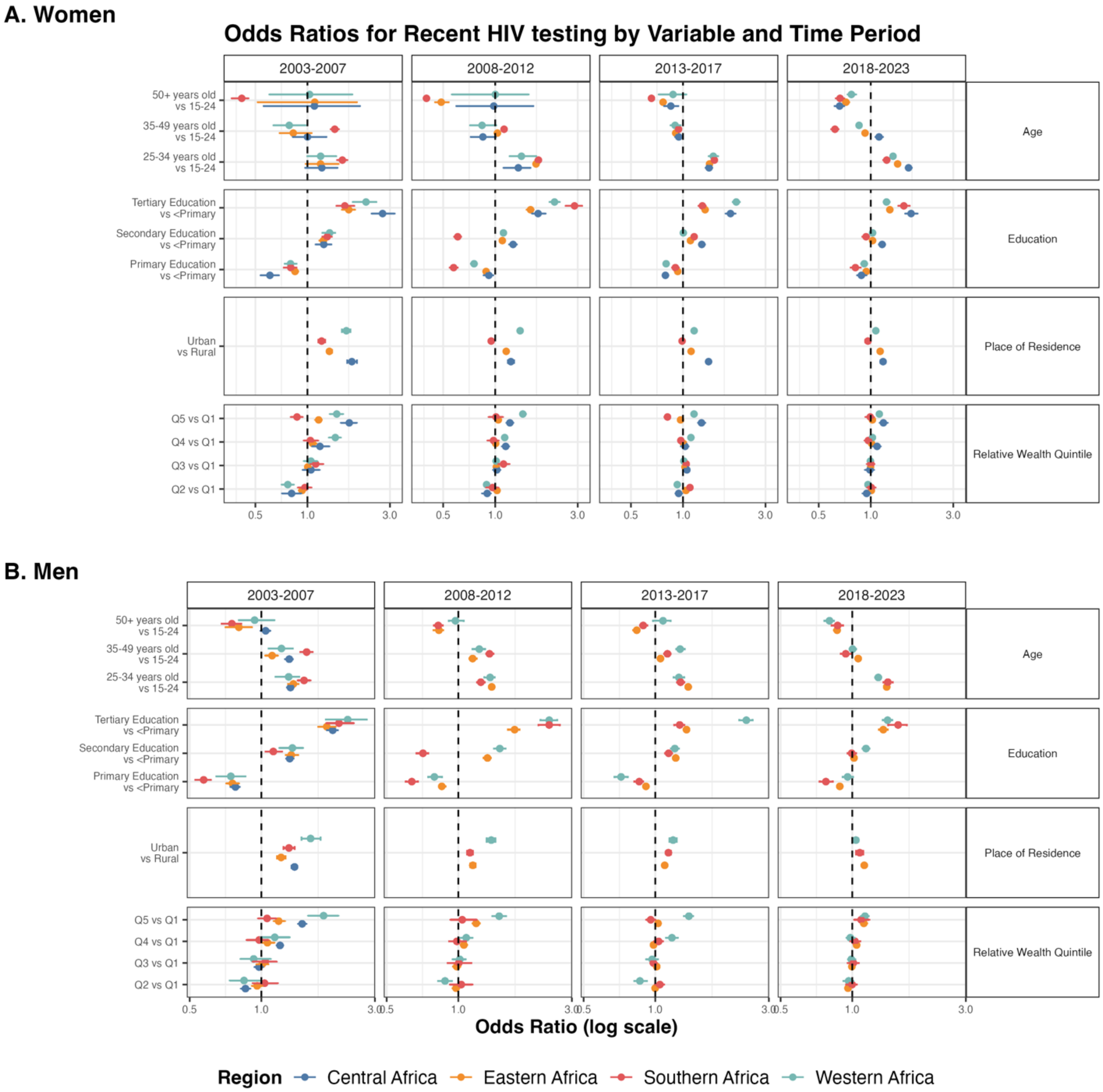

Figure S2.6: Odds-ratio of recent HIV testing, by factor, time-period, and sex. This figure shows the estimated odds-ratio derived from a hierarchical regression model of recent HIV testing (defined as having tested for HIV and received the results in the 12 months preceding the survey) for the four socio-demographic covariates, age, place of residence, education and relative wealth quintile, among women (A), and men (B). The mean effect estimates are depicted as circles. Horizontal lines are 95% credible intervals. The model was stratified by sex, and regions of sub-Saharan Africa. Effect estimates for each region are presented in different colors.

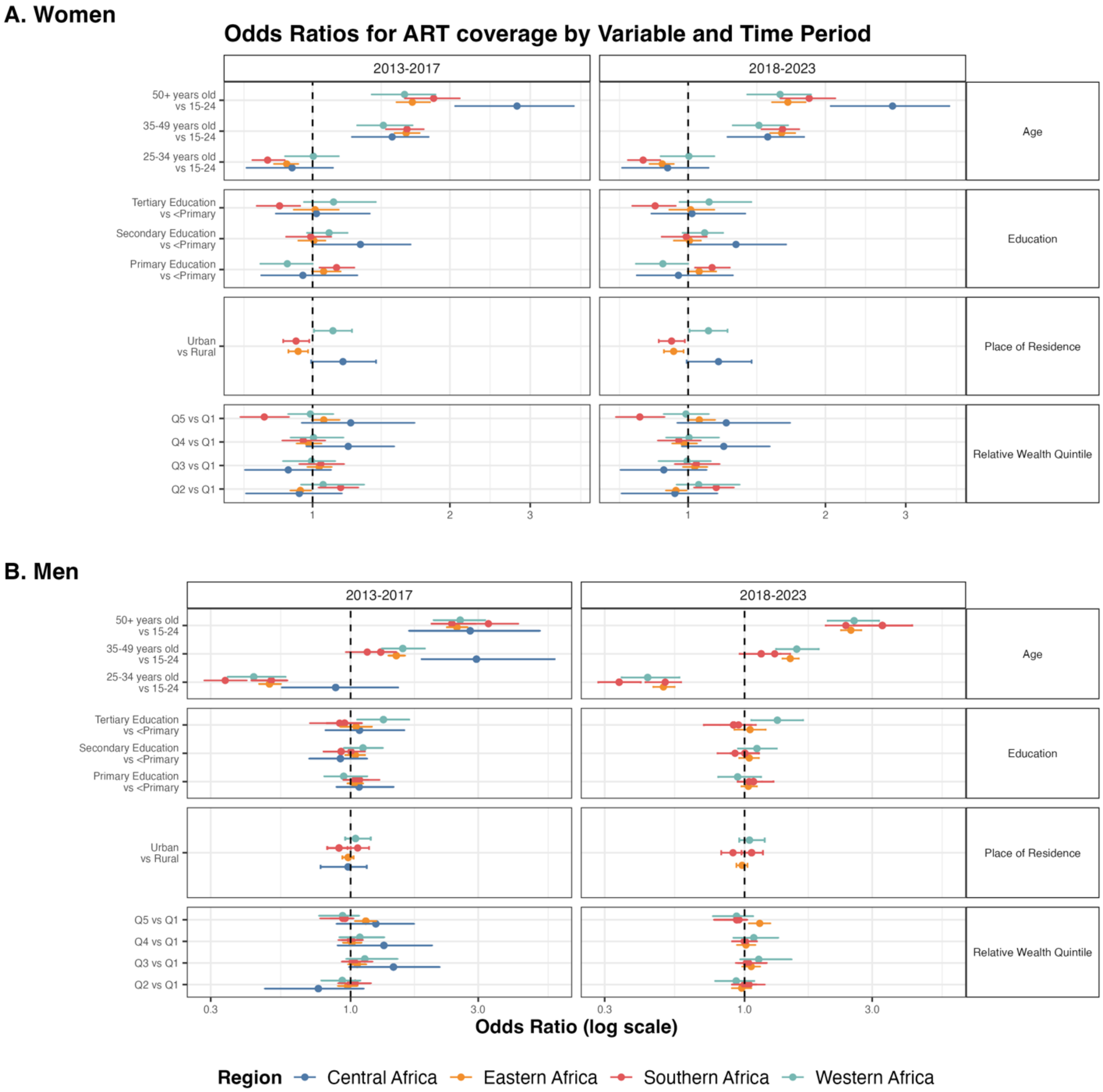

Figure S2.7: Odds-ratio of ART coverage among PLHIV, by factor, time-period, and sex. This figure shows the estimated odds-ratio derived from a hierarchical regression model of ART coverage among people living with HIV (PLHIV) for the four socio-demographic covariates, age, place of residence, education and relative wealth quintile, among women (A), and men (B). The mean effect estimates are depicted as circles. Horizontal lines are 95% credible intervals. The model was stratified by sex, and regions of sub-Saharan Africa. Effect estimates for each region are presented in different colors.

Model Selection Results

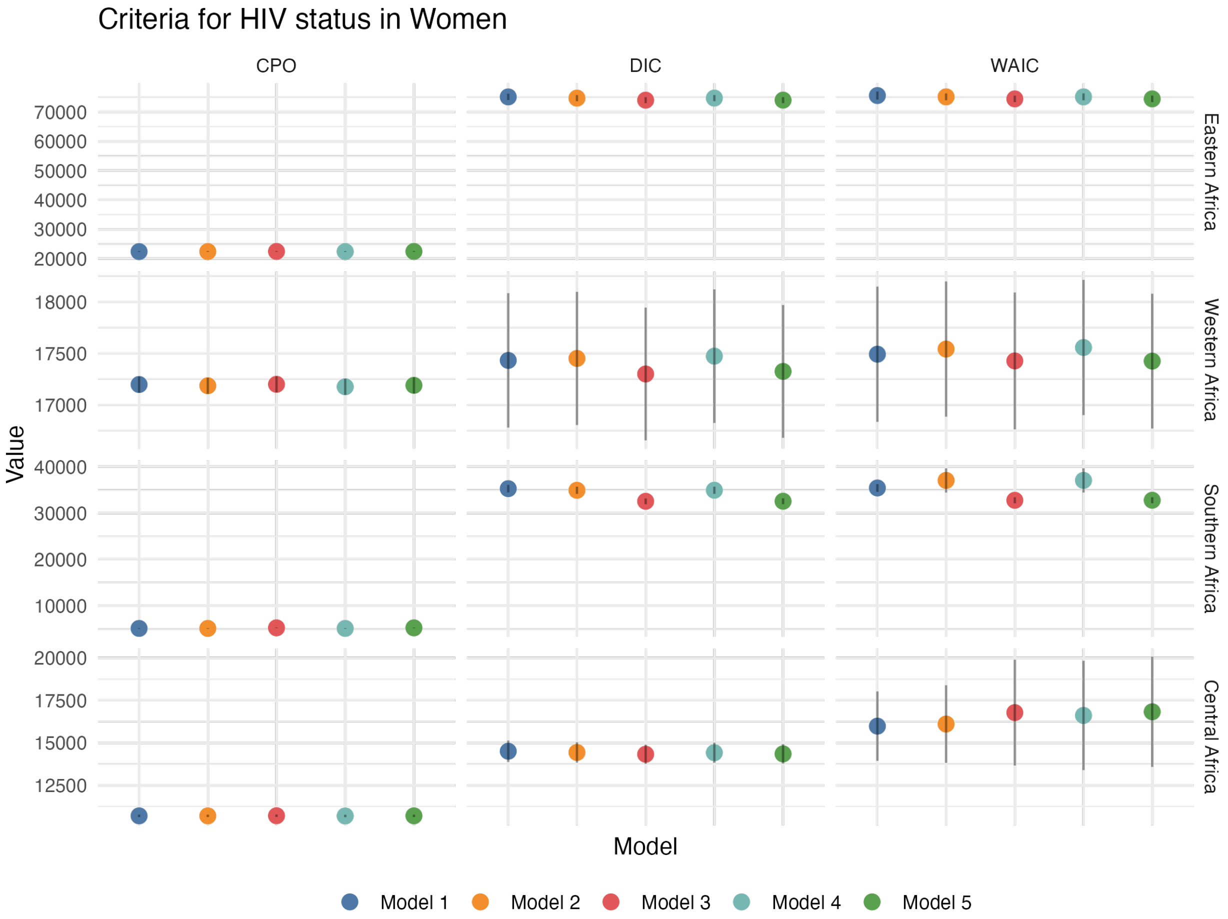

Figure S2.8: Performance for the logistic regression model of HIV status among women, for the five model formulations, according to CPO, DIC, and WAIC. This figure shows the values of the CPO, DIC, and WAIC, for the five model formulations across Eastern, Western, Southern, and Central Africa. For the CPO, higher values indicate better model performance while for the DIC and WAIC, lower values indicate better model performance. Circle represents mean estimates, while vertical bars represent uncertainty associated with the corresponding criterion.

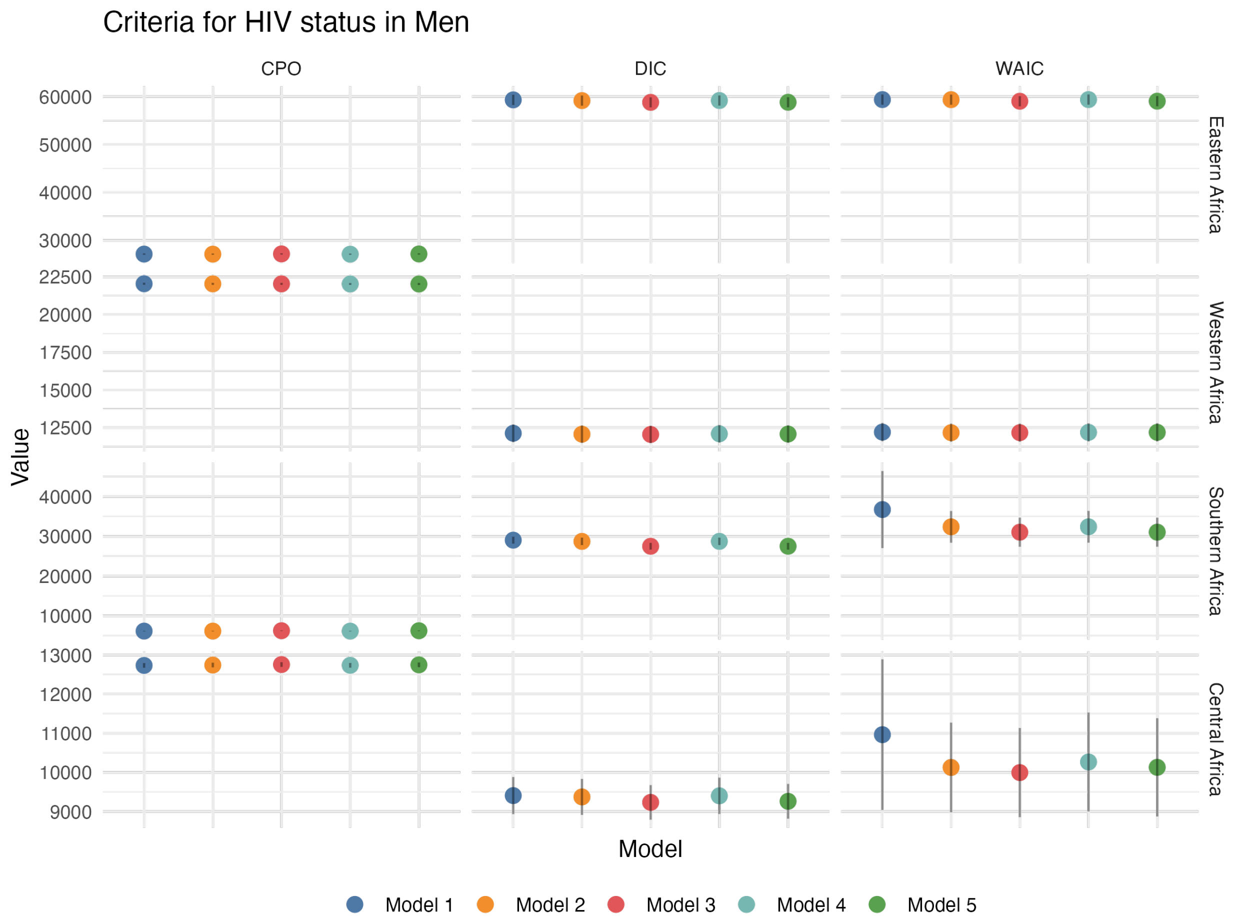

Figure S2.9: Performance for the logistic regression model of HIV status among men, for the five model formulations, according to CPO, DIC, and WAIC. See figure S2.7 for more details.

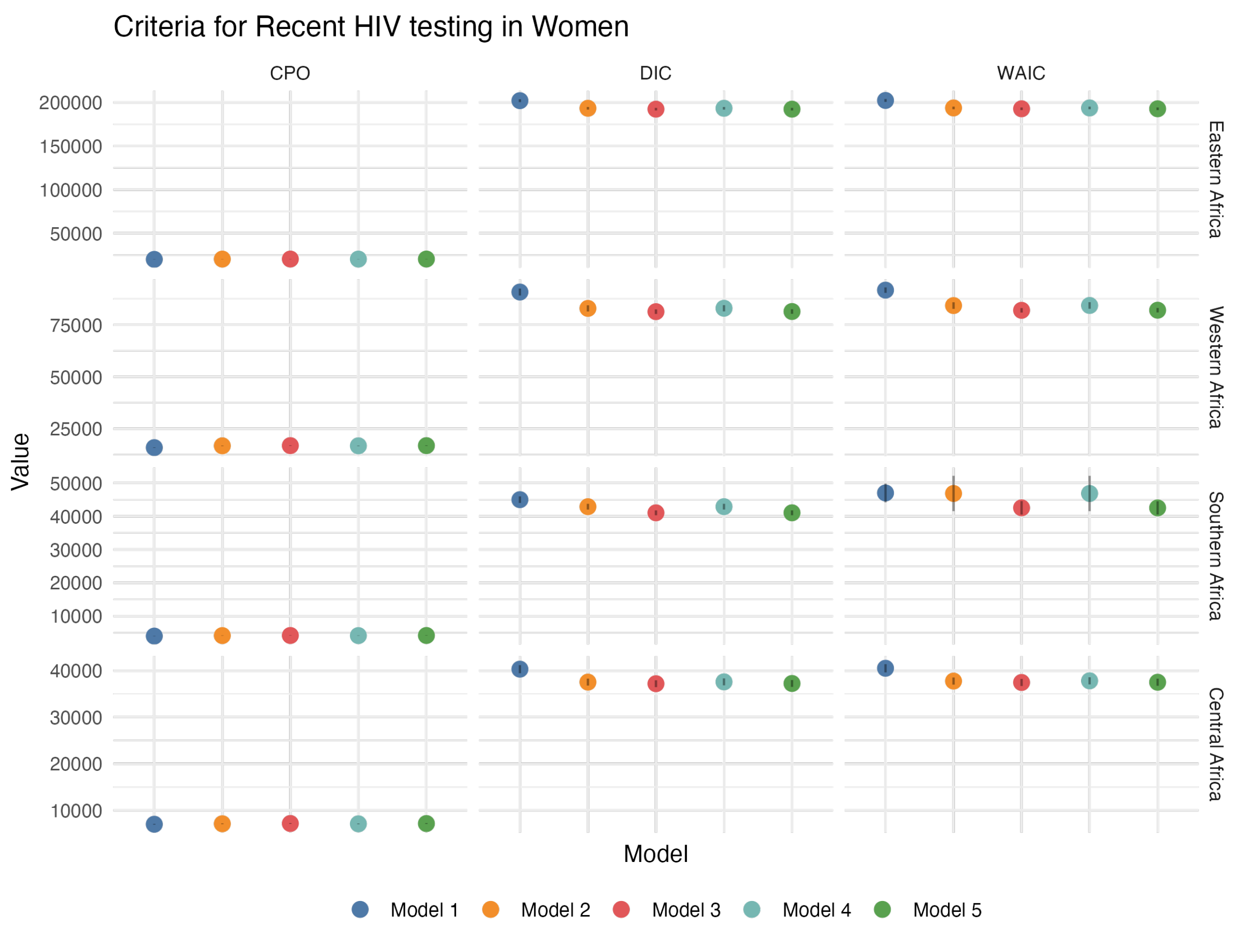

Figure S2.10: Performance for the logistic regression model of recent HIV testing among women, for the five model formulations, according to CPO, DIC, and WAIC. See figure S2.7 for more details.

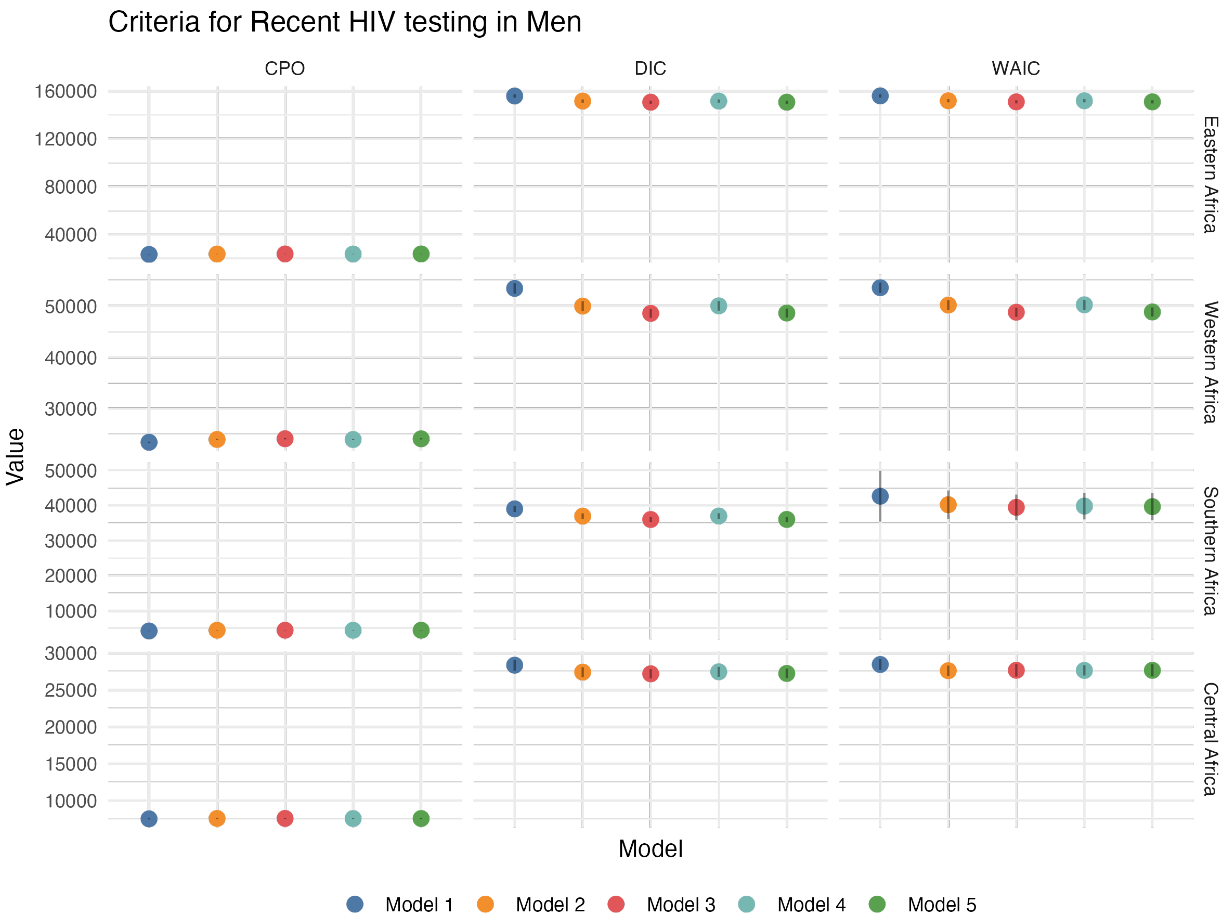

Figure S2.11: Performance for the logistic regression model of recent HIV testing among men, for the five model formulations, according to CPO, DIC, and WAIC. See figure S2.7 for more details.

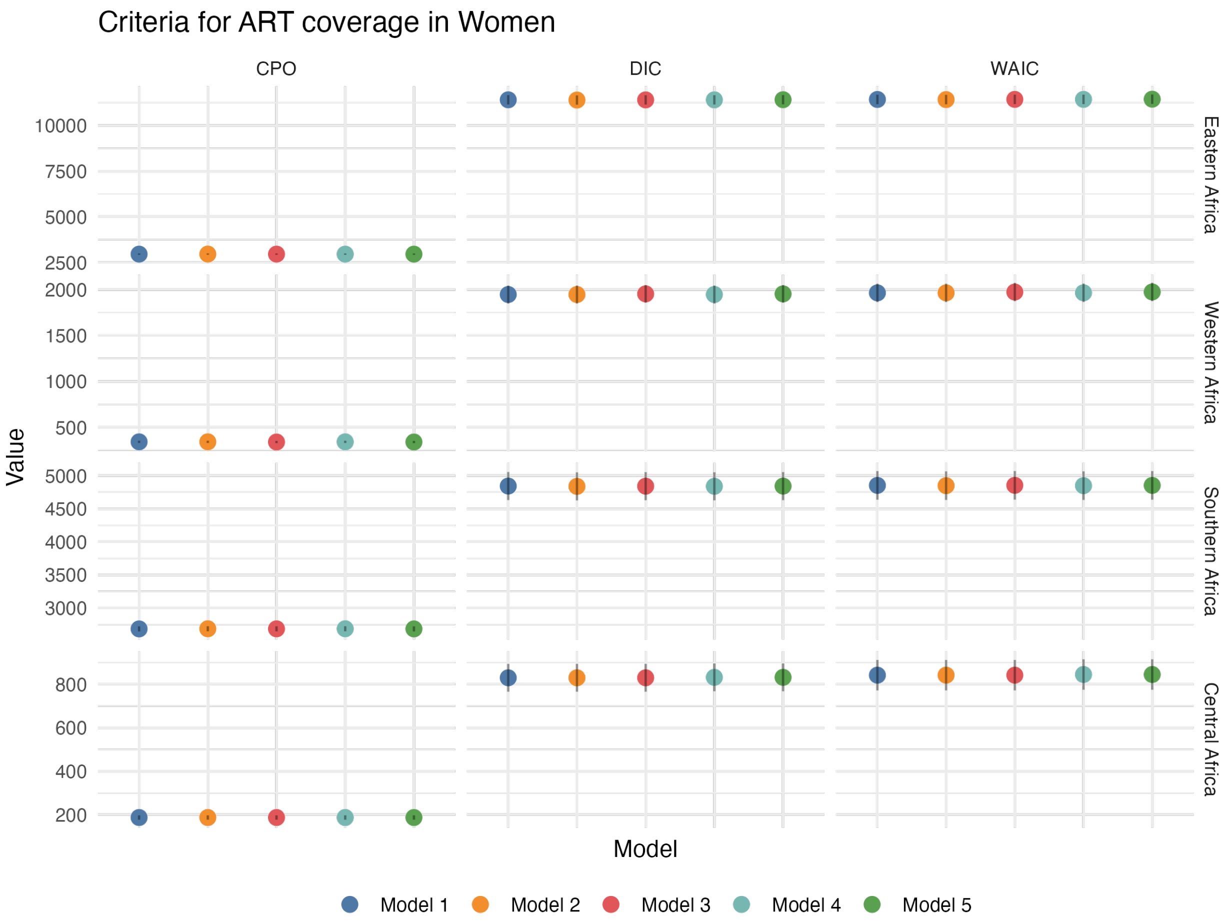

Figure S2.12: Performance for the logistic regression model of ART coverage among women living with HIV, for the five model formulations, according to CPO, DIC, and WAIC. See figure S2.7 for more details.

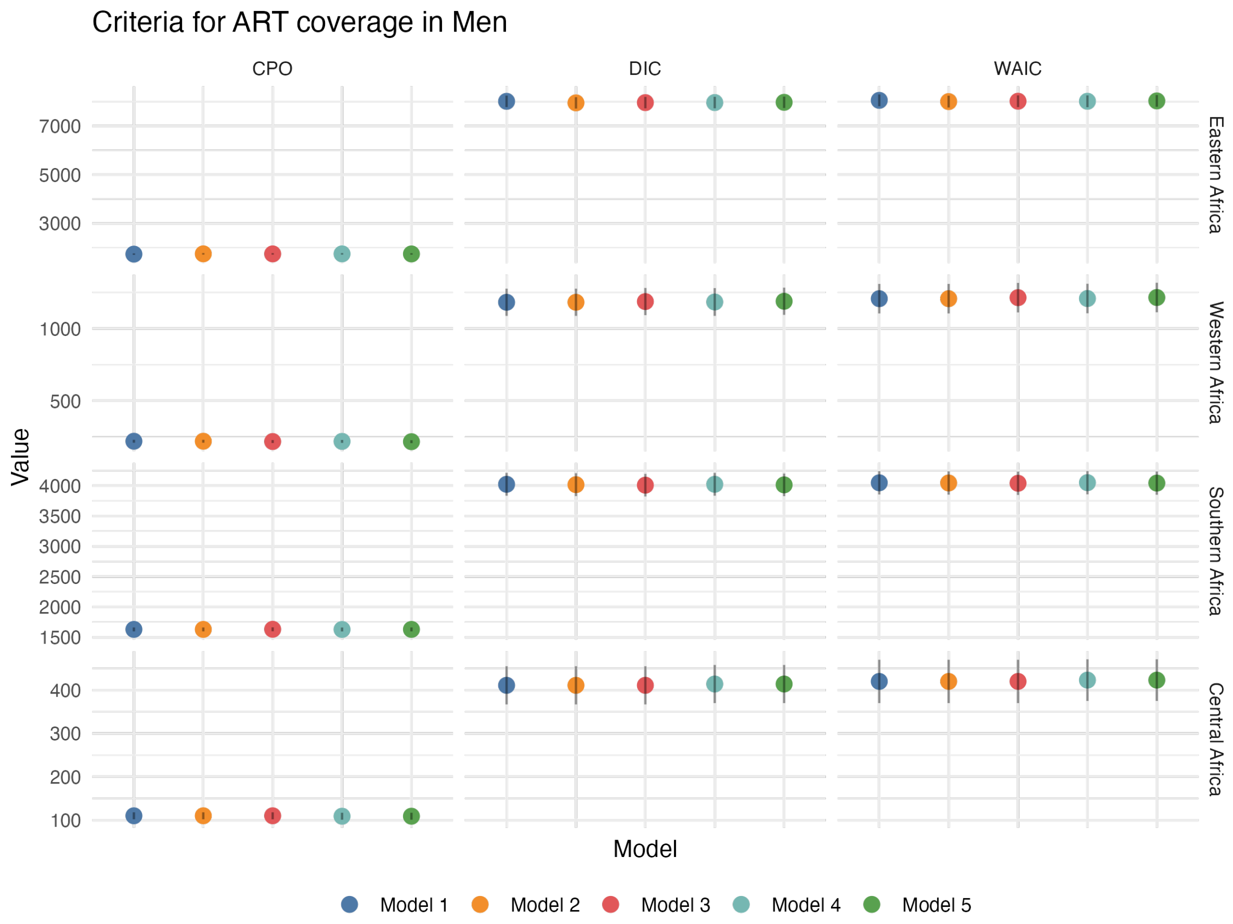

Figure S2.13: Performance for the logistic regression model of ART coverage among men living with HIV, for the five model formulations, according to CPO, DIC, and WAIC. See figure S2.7 for more details.

Sensitivity analyses

Spearman’s rank correlation coefficient

To assess potential multicollinearity between education and relative wealth, we calculated the Spearman’s rho coefficients between these variables across all surveys. The median rho was 0.31 (range: 0.07 to 0.51), indicating a low to moderately strong positive association. This suggests that multicollinearity is not a significant concern, and that education and wealth contribute unique information to the model.

Difference in Average Predictive Comparisons

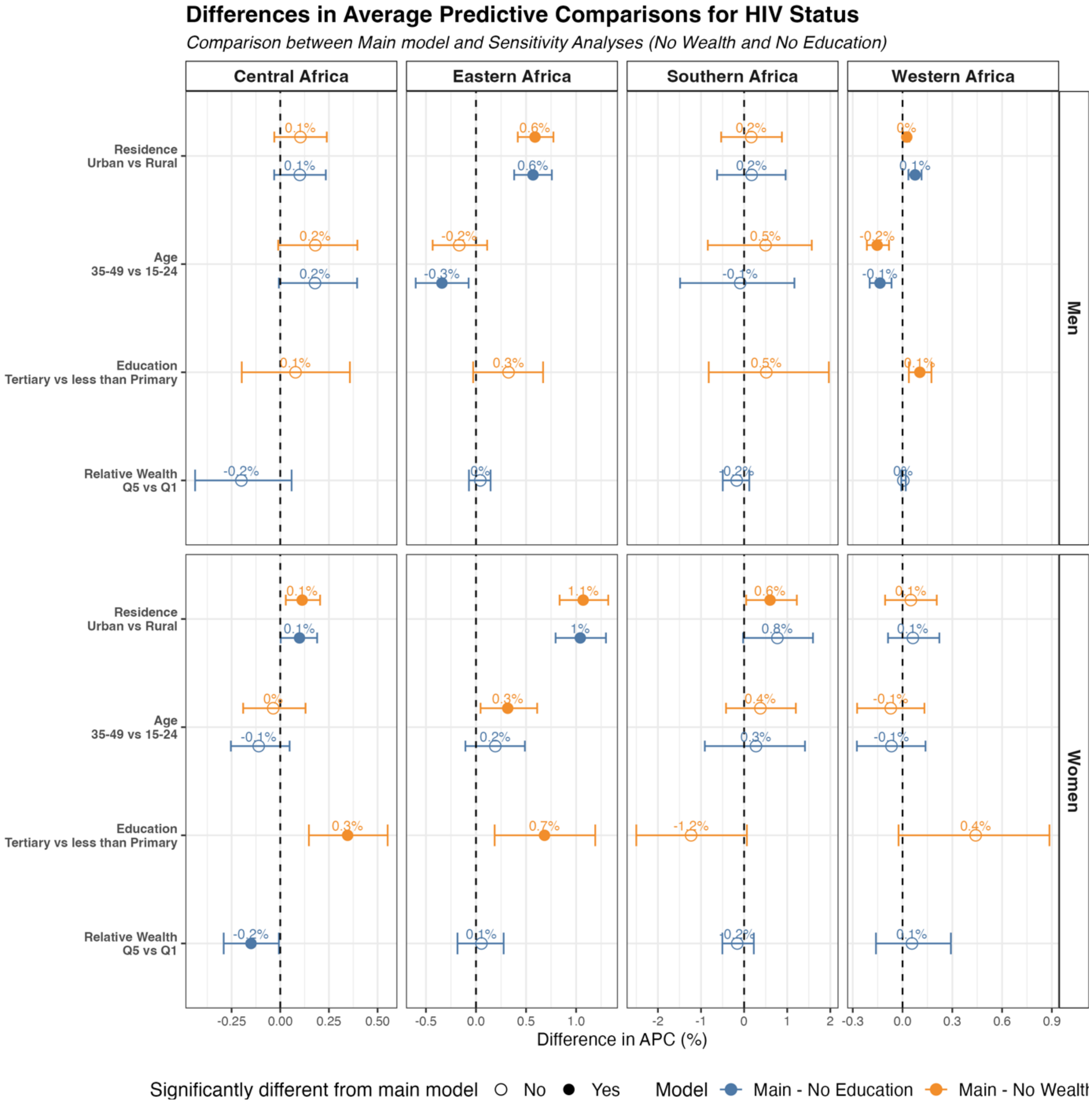

Figure S2.14: Differences in Average Predictive Comparisons for HIV status between main model and sensitivity analyses. This figure displays the differences in Average Predictive Comparisons (APCs) between the Main model and the sensitivity analyses (No Wealth, in orange, and No Education models, in blue) for ART Coverage (antiretroviral therapy uptake among HIV-positive individuals). The mean effect estimates are depicted as circles. Horizontal lines are 95% credible intervals. The model was stratified by sex, and regions of sub-Saharan Africa.

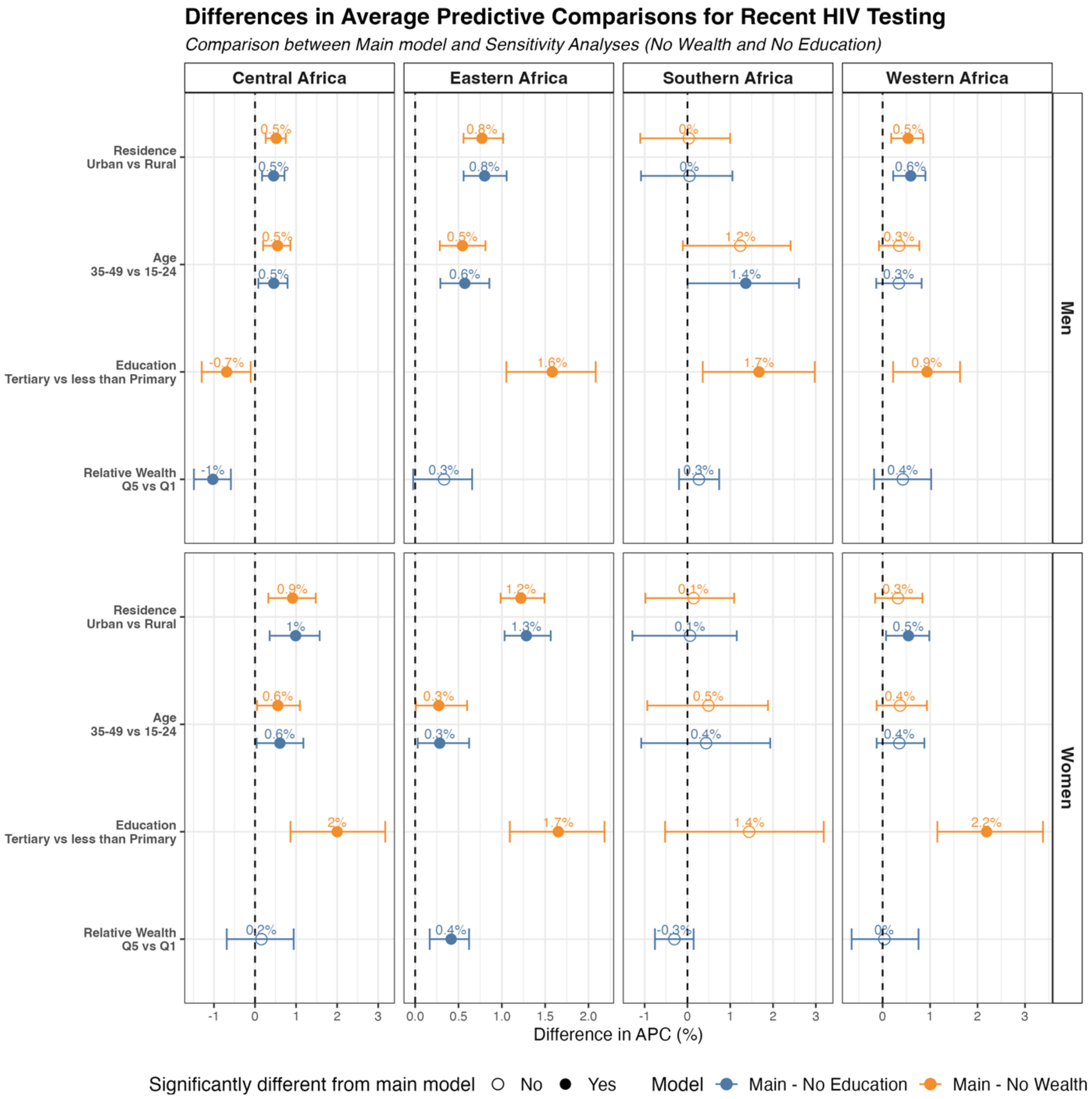

Figure S2.15: Differences in Average Predictive Comparisons for recent HIV testing between main model and sensitivity analyses. See figure S2.13 for more details.

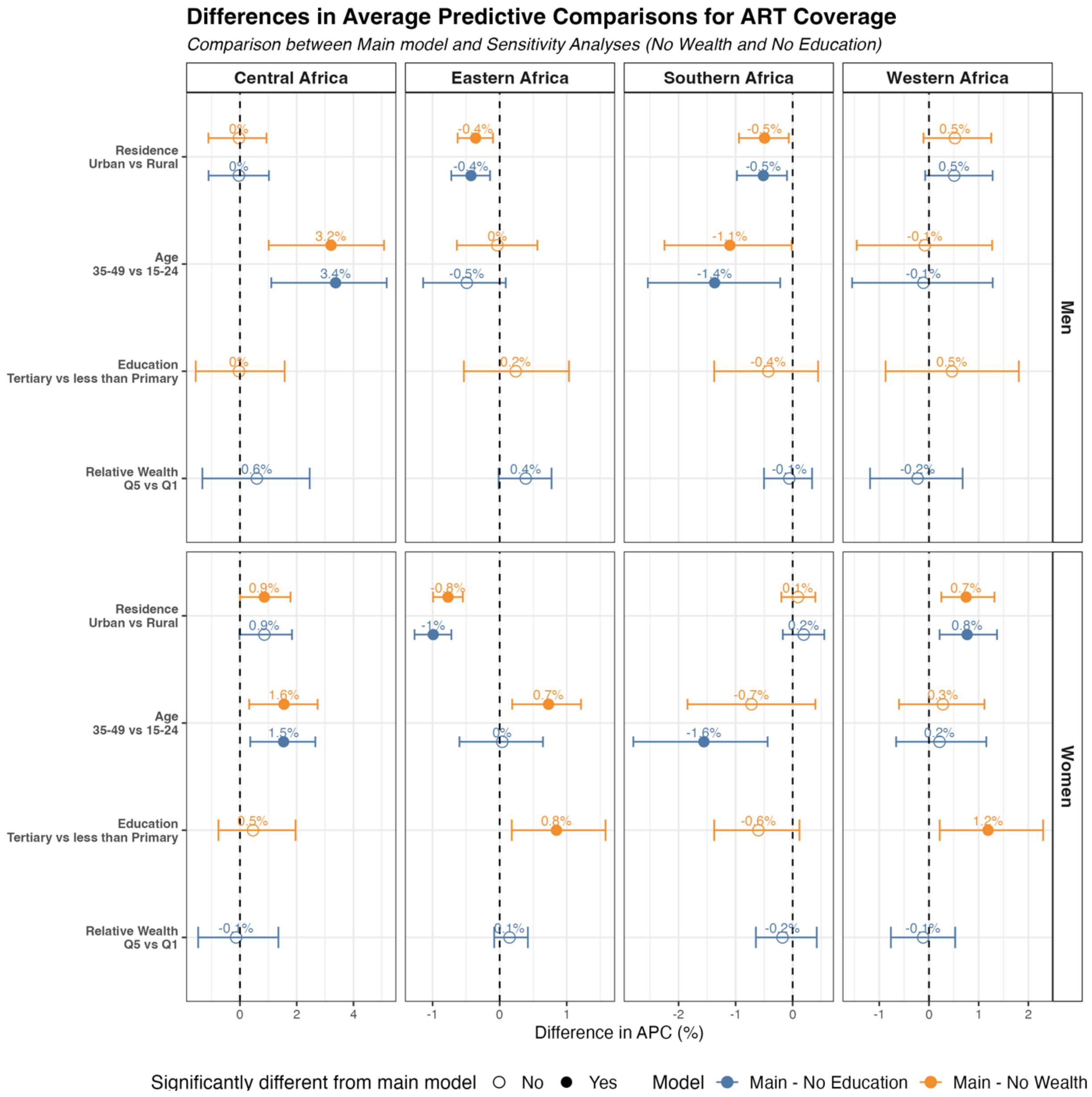

Figure S2.16: Differences in Average Predictive Comparisons for ART coverage among people living with HIV between main model and sensitivity analyses. See figure S2.13 for more details.
